# Positional Cues in the *Drosophila* Nerve Cord: Semaphorins Pattern the Dorso-Ventral Axis

**DOI:** 10.1371/journal.pbio.1000135

**Published:** 2009-06-23

**Authors:** Marta Zlatic, Feng Li, Maura Strigini, Wesley Grueber, Michael Bate

**Affiliations:** 1 Department of Zoology, University of Cambridge, Cambridge, United Kingdom; 2 Department of Physiology and Cellular Biophysics, Columbia University, New York, New York, Unites States of America; 3 Howard Hughes Medical Institute (HHMI) Janelia Farm Research Campus, Ashburn, Virginia, United States of America; 4 Institute of Molecular Biology and Biotechnology (IMBB)-FORTH, Iraklio, Crete, Greece; Stanford University, United States of America

## Abstract

During the development of neural circuitry, neurons of different kinds establish specific synaptic connections by selecting appropriate targets from large numbers of alternatives. The range of alternative targets is reduced by well organised patterns of growth, termination, and branching that deliver the terminals of appropriate pre- and postsynaptic partners to restricted volumes of the developing nervous system. We use the axons of embryonic *Drosophila* sensory neurons as a model system in which to study the way in which growing neurons are guided to terminate in specific volumes of the developing nervous system. The mediolateral positions of sensory arbors are controlled by the response of Robo receptors to a Slit gradient. Here we make a genetic analysis of factors regulating position in the dorso-ventral axis. We find that dorso-ventral layers of neuropile contain different levels and combinations of Semaphorins. We demonstrate the existence of a central to dorsal and central to ventral gradient of Sema 2a, perpendicular to the Slit gradient. We show that a combination of Plexin A (Plex A) and Plexin B (Plex B) receptors specifies the ventral projection of sensory neurons by responding to high concentrations of Semaphorin 1a (Sema 1a) and Semaphorin 2a (Sema 2a). Together our findings support the idea that axons are delivered to particular regions of the neuropile by their responses to systems of positional cues in each dimension.

## Abstract 2

Positional cues target sensory axons to appropriate volumes of the developing nervous system independently of their synaptic partners.

## Author Summary

Axons and dendrites of synaptic partners must be targeted to a common region of the developing neural network so that appropriate connections can be formed. The mechanisms underlying this targeting are incompletely understood. We showed previously that a positional cue (Slit) acting in the medio-lateral axis of the *Drosophila* nerve cord controls the position of sensory terminals independently of their synaptic partners. This work revealed that there might be additional cues operating in a similar fashion in the dorso-ventral axis of the nerve cord. Here we report the discovery of a dorso-ventral system of positional cues, in the form of a gradient of secreted Semaphorin 2a acting at right angles to the Slit gradient, and membrane bound Semaphorin 1a differentially distributed across the neuropile. The two Semaphorins dictate the termination positions of sensory axons in the dorso-ventral axis. Together with a third signal acting in the antero-posterior axis, Semaphorins and Slit deliver axons to appropriate volumes of the neural network. These studies support a model in which axons branch and terminate, independently of synaptic partners, in response to pervasive systems of volumetric positional cues.

## Introduction

During the development of neural circuitry, neurons of different kinds must establish specific synaptic connections by selecting appropriate targets from large numbers of different alternatives. The range of these alternative targets is reduced by well organised patterns of growth, termination, and branching that deliver the terminals of appropriate pre- and postsynaptic partners to restricted regions of the developing nervous system. The mechanisms that control the coordinate projection of pre- and postsynaptic neurites to a common region are incompletely understood. Although there has been substantial progress in identifying molecular mechanisms of axon growth and guidance, far less is known about the way in which appropriate target areas are identified, leading to termination and branching [1]–[4]. The extent to which these processes depend on target specific signals as opposed to pervasive guidance cues, to which many different neurons can respond, is far from clear.

**Figure 1:**
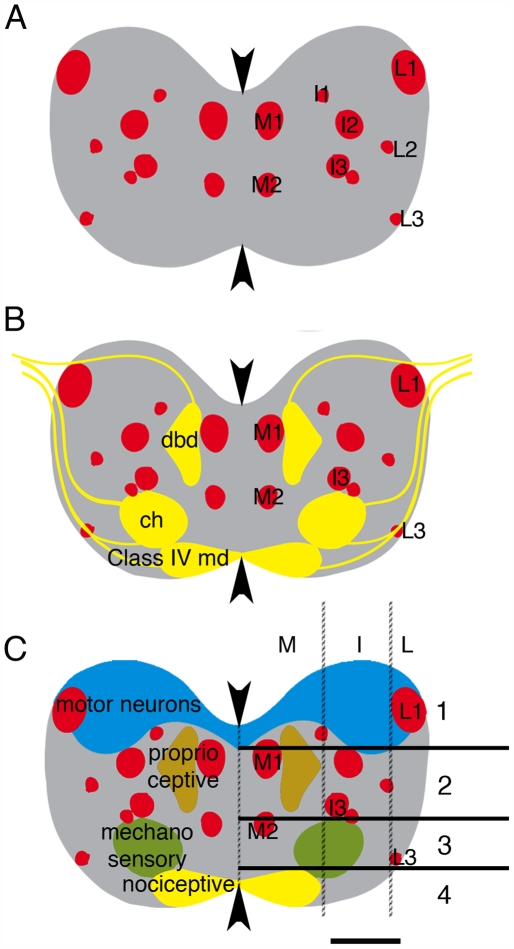
Different neuron classes project to different medio-lateral domains and to different dorso-ventral layers of neuropile. (A–C) Diagrams of neuropile in 21-h embryos in transverse section. Each diagram represents a projection of a confocal z series through an abdominal segment. Dorsal up. Arrowheads show midline. (A) Distribution of Fas II tracts (red) in neuropile (grey). Fas II tracts are named after medial, intermediate, and lateral domains of the neuropile and ordered in the dorso-ventral axis by number (1 being the most dorsal of the tracts in a domain). There are two tracts (M1 and M2) in the medial, three in the intermediate (I1, I2 and I3), and three (L1, L2 and L3) in the lateral domain. Of these, M1, M2, I2, I3, and L1 are most prominent and can be used as reference points. (B) Projections (yellow) of dbd, ch, and class IV md neurons with respect to Fas II tracts (red). Dbd axons enter neuropile above L1 and grow through dorsal neuropile to M1 where they branch. Their terminals extend from M1 to the level of I3. Ch axons contact the neuropile initially at the level of L1 and turn sharply downwards, avoiding dorsal neuropile. They enter neuropile either at the level of the bottom of I3 (ISN ch axons) or at the level of L3 (SN ch axons). Ch axons branch and terminate underneath I3. Class IV md axons terminate in the most ventral and medial portion of neuropile. (C) Diagram of the four layers of neuropile, which differ in the afferent input they receive. Filled lines indicate dorso-ventral layer boundaries. Hatched lines indicate arbitrary domain boundaries in the medio-lateral axis. Layer 1 is where most motor neuron dendrites (blue) arborize and has little sensory input; layer 2 is where proprioceptive sensory neurons, including dbd terminate; layer 3 is where mechanosensory ch neurons terminate; layer 4 is where class II–IV md neurons terminate. The top of I2 marks the lower boundary of layer 1, the bottom of I3 marks the lower boundary of layer 2, and the bottom of L3 marks the lower boundary of layer 3. The boundary between layers 3 and 4 can also be defined as mid way between the ventral margin of the neuropile and the lower surface of M2, if L3 is not clearly visible.

We have used the axons of embryonic *Drosophila* sensory neurons as a model system in which to study the way in which growing neurons are guided to terminate in a specific region of the developing nervous system. These neurons have their cell bodies in the periphery of the embryo, either close to or embedded in the body wall. Their axons grow into a central ganglion where they terminate in a neuropile that consists of a dense meshwork of interweaving axons and dendrites. Anatomically the neuropile shows few overt signs of organisation apart from clear regularities such as the commissures that cross the midline and a set of longitudinal axon bundles at stereotyped positions that provide a series of landmarks with respect to which other structures can be mapped [5]. Functionally however the neuropile is an obviously well-organised structure, with, for example, motor neuron dendrites and the endings of sensory neurons terminating and branching in distinct and characteristic domains. Thus it is clear that there must be cues operating in the neuropile that deliver terminals to these specific destinations within the forming network. In the case of the sensory neurons, it is clear that specific types of neurons serving particular modalities terminate in well ordered and characteristically different parts of the neuropile. These termination zones together with the overall structure of the neuropile are shown in diagrammatic form in Figure 1. Because the sensory neurons provide us with an accessible set of cells whose terminals grow to different parts of the forming neuropile, we can readily use these neurons to investigate the guidance mechanisms that operate to determine these distinctive patterns of growth and termination.

We previously showed that Slit secreted at the midline and acting through its Robo receptors constitutes a repellent gradient to which sensory neurons respond by terminating and branching at specific positions in the medio-lateral axis of the neuropile [6]. Expression of a particular Robo receptor by a sensory axon is necessary and sufficient to determine the distance from the midline at which that axon will terminate. Thus, in the medio-lateral axis at least, the position at which an axon terminates within the forming neuropile is determined not by some putative signals from its postsynaptic target, but by the presynaptic neuron's response to a pervasive cue secreted from the midline. However, the neuropile is a 3-D structure and there must therefore be additional cues that determine the dorso-ventral and antero-posterior termination domains for each axonal and dendritic arbor.

Our previous study provided evidence for at least one further signal that operates to determine positions in the dorso-ventral axis. Sensory terminals that are shifted experimentally along the medio-lateral axis of the neuropile maintain their characteristic dorso-ventral location in their new position, suggesting that the factor that determines this position may be a “dorso-ventral” patterning cue that is present at different positions in the medio-lateral axis. This additional finding led us to propose a general model for the cues that delineate domains within a neuropile in which presynaptic axons and their postsynaptic partners terminate and form connections [6]. In this model, termination sites depend on the response of axons to a system of positional cues that dictate the behaviour and final location of many, perhaps all terminals within a developing network of pre- and postsynaptic neurons. Specific locations are given not by the target, but by the set of receptors for these positional cues that each neuron expresses.

Here, we test and augment this model by using a genetic screen to identify cues and their receptors that guide terminating axons in the dorso-ventral axis of the neuropile. We find that dorso-ventral layers of neuropile contain different levels and combinations of semaphorins. We demonstrate the existence of a central to dorsal and central to ventral gradient of Sema 2a, perpendicular to the Slit gradient. We show that a combination of Plexin A (Plex A) and Plexin B (Plex B) receptors specifies the ventral projection of sensory neurons by responding to high concentrations of Semaphorin 1a (Sema 1a) and Semaphorin 2a (Sema 2a). These signals together with the Slit/Robo system acting in the medio-lateral axis limit the arborisations of sensory axons to specific *termination domains* within the neuropile. Since these are the domains within which specific functional sets of connections will be formed, the terminating sensory axons, by responding to pervasive positional cues, are able to lay out part of the characteristic functional architecture of the forming network.

## Results

### The *Drosophila* Embryonic Neuropile Can Be Divided into Four Dorso-Ventral Layers, Which Are Likely to Have Distinct Functions in Information Processing

Previous studies have shown that the axons of sensory neurons project to distinct medio-lateral, dorso-ventral, and antero-posterior domains in the neuropile in correlation with their modality and dendritic morphology [7]–[9].

We have extended these studies using Fasciclin II (Fas II) positive tracts as reference points (Figure 1A) [6],[10]. We divide the neuropile into three medio-lateral domains and four dorso-ventral layers (Figure 1C). With the exception of the chordotonal (ch) neurons, sensory axons terminate in the medial domain of the neuropile. Ch axons terminate and branch in the intermediate domain. There is very little sensory input to the dorsal-most layer (layer 1) where motor neurons establish their dendritic arbors. The proprioceptive dorsal bipolar dendritic (dbd) and class I md (multidendritic) neurons terminate in the upper central layer (layer 2) [6],[9],[11]. The ch neurons terminate in the lower central layer (layer 3) [6], whereas nociceptive class IV md neurons terminate in the ventral-most layer (layer 4). Class IV md neurons can be identified with *ppkEGFP,* which labels one intersegmental nerve (ISN) and two segmental nerve (SN) neurons in each hemisegment [9],[12].

The position of termination in the neuropile does not correlate with the nerve route by which the sensory neurons reach the neuropile (see Figure S1). Sensory axons whose cell bodies are located ventrally in the body wall travel in the SN, whereas axons whose cell bodies are located dorsally or laterally in the body wall travel in the ISN [13]. Sensory axons running in the SN and ISN terminate in layers 2, 3, or 4, in correlation with their modality and dendritic morphology [9],[11]. Since each of the three modality-specific sensory termination domains contains some neurons that have travelled through the SN, and others that have travelled through the ISN, differences in axon routing to the neuropile cannot account for differences in termination within the neuropile.

### Expressing *plex B* or *plex A* in Sensory Neurons Shifts Their Terminals away from Central and Dorsal Layers of the Ventral Nerve Cord

To investigate mechanisms that confine sensory projections of different modalities to different dorso-ventral layers of the neuropile, we carried out a gain-of-function screen for trans-membrane proteins, which, when expressed selectively in sensory neurons, shift sensory terminals with respect to Fas II tracts.

**Figure 2:**
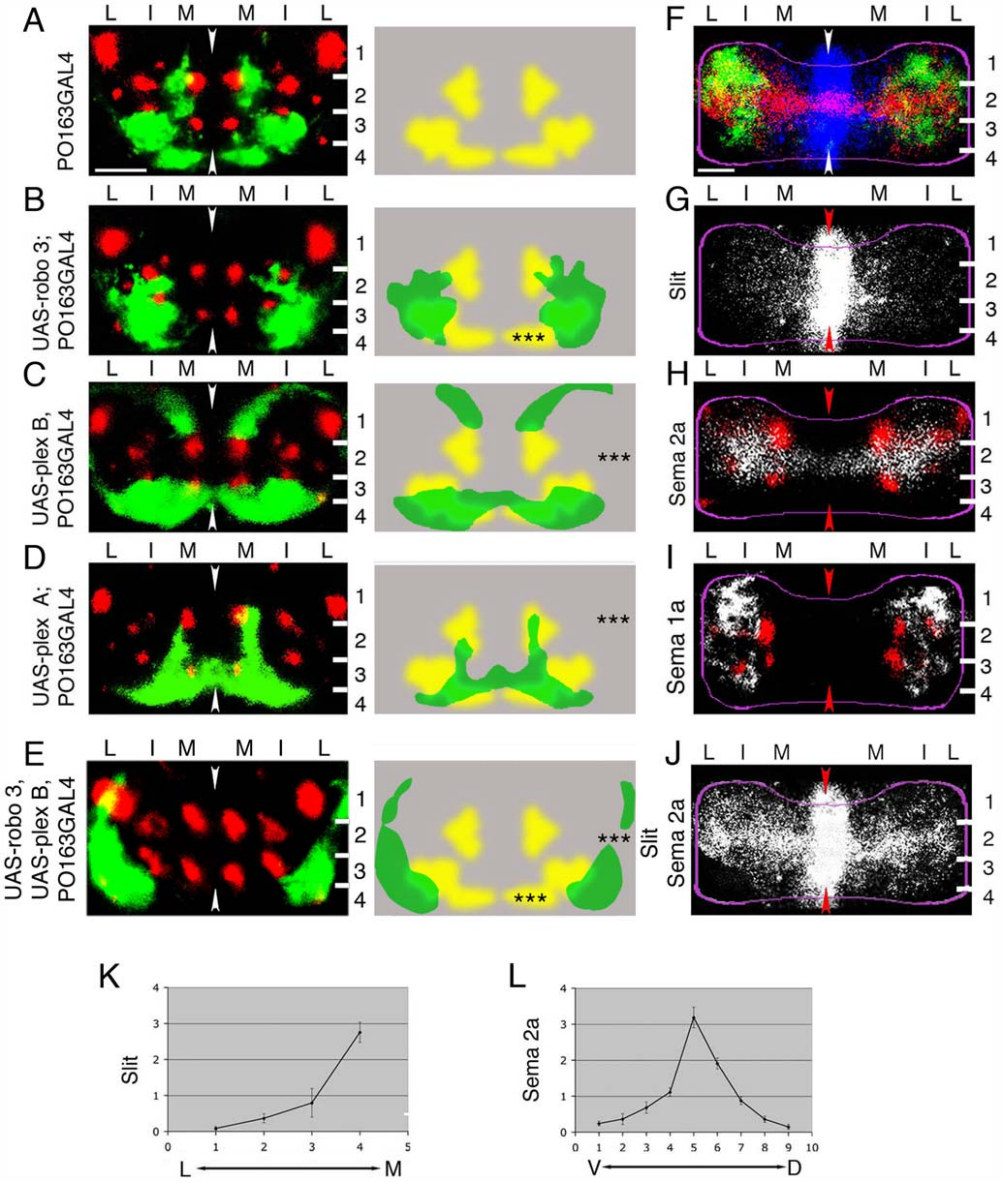
Effects of altering levels of receptors for Slit, Sema 2a, or Sema 1a in sensory neurons and the distribution of cues in neuropile. (A–E) Representative images of sensory terminals labelled with *PO163GAL4, UAS-n-syb-GFP* (green) with respect to Fas II tracts (red) in 21-h embryos (left) and diagrams showing patterns of sensory terminals superimposed on wild-type pattern (right). In all cases images show projections of a confocal z series of transverse sections through A7. Dorsal is up. Arrowheads show midline. White lines, layer boundaries. Numbers indicate layers. M, medial; I, intermediate; L, lateral domains. *, *p*<0.05; **, *p*<0.01; ***, *p*<0.001. Scale bar: 10 µm. (A) Wild-type pattern of sensory terminals revealed in A7 in the CNS of *PO163GAL4, UAS-n-syb-GFP* embryos. (B) Expressing Robo3 in sensory neurons excludes sensory terminals from the medial domain of neuropile. Sensory terminals shift laterally with respect to Fas II tracts. Quantification of the normalised surface area occupied by sensory terminals (sensory area [SA]) in the medial domain (*SA*_M/T_ = *SA*[medial]/*SA*[medial+intermediate+lateral]) reveals a significant decrease (***, *p* = 9×10^−25^; Student's *t*-test; average *SA*_M/T_ = 0.04; SD = 0.04; *n* = 30 hemisegments) with respect to wild-type embryos (average *SA*_M/T_ = 0.3; SD = 0.07; *n* = 30 hemisegments). (C) Expressing Plex B in sensory neurons results in exclusion of sensory neuron terminals from neuropile layer 2. Quantification of SA in layer 2 (*SA*_2/h_ = *SA*(layer 2)/[hemisegment surface area]) reveals a significant decrease (***, *p* = 4×10^−17^; Student's *t*-test; average *SA*_2/h_ = 0.003; SD = 0.006; *n* = 32 hemisegments) with respect to wild-type embryos (average *SA*_2/h_ = 0.06; SD = 0.02; *n* = 32 hemisegments). (D) Expressing Plex A in sensory neurons results in their exclusion from layer 1 and from intermediate portions of layer 3. Sensory terminals appear compressed into the most medial portion of layers 4, 2, and 3, so that the overall effect is a ventral/medial projection pattern in the form of an arc on both sides of the midline. Quantification of *SA*_1+3+4/h_ (*SA*_1+3+4/h_ = *SA*[layer 1+3+4]/[hemisegment surface area]) reveals a significant decrease (***, *p* = 2×10^−22^; Student's *t*-test; average *SA*_1+3+4/h_ = 0.1; SD = 0.04; *n* = 38 hemisegments) with respect to wild-type embryos (average *SA*_1+3+4/h_ = 0.3; SD = 0.05; *n* = 32 hemisegments). (E) Co-expressing Robo3 and Plex B in sensory neurons produces a “combination” of Robo 3 and Plex B expression phenotypes. Sensory terminals are now mostly confined to the lateral-most portion of layers 3 and 4. Quantification of SA in the medial domain reveals a significant decrease (***, *p* = 1×10^−14^; Student's *t*-test; average *SA*_M/T_ = 0.03; SD = 0.05; *n* = 14 hemisegments) with respect to wild-type embryos (average *SA*_M/T_ = 0.3; SD = 0.07; *n* = 30 hemisegments). Quantification of SA in layer 2 reveals a significant decrease (***, *p* = 6×10^−15^; Student's *t*-test; average *SA*_2/h_ = 0.004; SD = 0.009; *n* = 32 hemisegments) with respect to wild-type embryos (average *SA*_2/h_ = 0.06; SD = 0.02; *n* = 32 hemisegments). (F–I) Immunofluorescence visualisation of Slit, Sema 2a, and Sema 1a (F, G, and J) and mapping of Sema 2a and Sema 1a (white) with respect to Fas II tracts (red) (H and I) in 13-h-old embryos. Images show projections of confocal z series of transverse sections through A7. Dorsal is up. Arrowheads show midline. White lines, layer boundaries. Magenta lines, neuropile outlines. Numbers indicate layers: M, medial; I, intermediate; L, lateral domains. Scale bar: 5 µm. (F) Superposition of Slit (blue), Sema 2a (red), and Sema 1a (green) patterns. (G) Slit is expressed at highest levels at the midline in all dorso-ventral layers of the neuropile. It forms a medial to lateral concentration gradient. (H and I) Mapping of Sema 2a and Sema 1a expression with respect to Fas II tracts. Note that pattern of forming Fas II tracts in 13-h embryos is variable and slightly different from that in 21-h embryos. However, prominent tracts are still readily recognisable reference points. (H) Sema 2a is expressed at high levels in a medio-lateral stripe perpendicular to the midline extending across the central region of neuropile in layer 2 between I2 and I3. It forms central to dorsal and central to ventral concentration gradients. Expression of the Plex B receptor for Sema 2a (see C) shifts sensory terminals away from high Sema 2a levels. (I) Sema1a expression is strongest in layer 1 and in the intermediate portions of layer 3. Sema 1a is very weakly if at all expressed in layer 4 and in the most medial parts of the neuropile. Expression of the Plex A receptor for Sema 1a (see D) results in the exclusion of sensory terminals from regions with high Sema 1a levels. (J) Superposition of Slit and Sema 2a (both white) patterns. Co-expression of Robo 3 and Plex B (see E) shifts sensory terminals from high Slit and Sema 2a levels. (K) Quantification of the Slit gradient from lateral (L) to medial (M) in a hemisegment (*n* = 12 hemisegments). Note the medial to lateral gradient. (L) Quantification of the Sema 2a gradient from the ventral (V) to dorsal (D) neuropile (*n* = 9). Note the central to ventral and the central to dorsal gradients.

We used *PO163GAL4*, *UAS-n-synaptobrevin-GFP* flies to target gene expression selectively to sensory neurons and simultaneously to visualise their terminals (Figure 2A) [14]. As a test of our method, we confirmed that expressing the Robo 3 receptor for Slit in sensory neurons shifts their terminals away from the medial domain of neuropile (Figure 2B) [6].

We screened 418 lines with UAS inserts in front of trans-membrane protein coding genes (see Materials and Methods and Table S1 for detailed results of the screen) by systematically expressing them in sensory neurons and analysing the pattern of sensory terminals in abdominal segments (A1–A7) at 21-h after egg laying (AEL).

We identified 11 genes (2.6%) that change the pattern of sensory terminals, without altering the number of neurons or preventing sensory axons from reaching the central nervous system (CNS) (Table S1). Of the 11 genes expressed, two produced obvious shifts along the dorso-ventral axis. Both belong to the same family: *plex B* and *A*. If *plex B* is expressed in all sensory neurons, sensory terminals are excluded from layer 2 (Figure 2C). If *plex A* is expressed, terminals are excluded from the intermediate regions of layer 3 and from layer 1 (Figure 2D). We also co-expressed Robo3 and Plex B in sensory neurons and found that this produces a “combination” of Robo 3 and Plex B expression phenotypes. In these embryos sensory terminals are now mostly confined to the lateral-most portion of layers 3 and 4 (Figure 2E).

### Sema 2a and Sema 1a Are Expressed in Central and Dorsal Layers of the Ventral Nerve Cord

The Plexins are receptors for the Semaphorins (Semas), a diverse family of secreted and membrane-associated proteins [15]–[19]. In *Drosophila* there are two Plexins (A and B) and five Semas: 1a, 1b, and 5c (transmembrane) and 2a and 2b (secreted). Plex B binds Sema 2a and mediates the Sema 2a-dependent repulsion of motor and sensory axons in the periphery and the fasciculation of longitudinal tracts in the ventral nerve cord (VNC) [20],[21]. Plex A binds strongly to Sema 1a and Sema 1b and mediates the Sema-dependent repulsion of embryonic motor axons in the periphery and the repulsion of adult olfactory receptor axons by Sema 1a in the antennal lobes [22]–[24].

The Plexin overexpression phenotypes suggested that their Sema ligands might act as cues to position the terminals of neurons along the dorso-ventral axis of the forming neuropile. We therefore used antibody labelling to analyse the expression of Semas 2a and 1a in the CNS at different stages of embryogenesis: prior to sensory axon ingrowth (11-h AEL), at stages when sensory axons form their terminal arbors (13-h AEL), and several hours after sensory axons have completed their terminal arbors (21-h AEL).

Sema 2a expression first becomes detectable at 11 h as the outgrowth of sensory axons begins, persists strongly until 16 h, but has disappeared by 21 h, when the embryo is mature and ready to hatch. At 13 h, when sensory axons are forming their terminal arbors, the highest levels of Sema 2a are in layer 2 in the centre of the neuropile (Figure 2F and 2H). Strikingly, the protein forms gradients of expression in the neuropile that extend dorsally and ventrally from layer 2 (Figure 2L), at right angles to the mediolateral gradient of Slit (Figure 2F, 2G, 2J, and 2K). There is no detectable expression in layer 4. Our experiments show that the effect of overexpressing Plex B in sensory neurons is to shift their terminals away from regions with high Sema 2a levels. This effect is still detectable at 21 h when there is no Sema 2a expression and we conclude that misplaced terminals do not compensate by delayed growth into central neuropile (Figure 2C).

Sema 1a expression is present at 10-h AEL, before sensory axons have entered the neuropile and persists throughout embryogenesis (unpublished data). By 13 h the highest levels of Sema 1a are in the lateral and intermediate portions of layers 1 and 3, at lower levels in layer 2, and not detectable in layer 4 (Figure 2F and 2I). In addition to differences in the levels of Sema 1a expression in different dorso-ventral layers of the neuropile, we also find an apparent decrease in concentration from intermediate (high) to medial (low) in layers 1 and 3. At 21 h Sema 1a is still strong in intermediate portions of layers 1 and 3. The effect of overexpressing Plex A is to exclude sensory terminals from these high levels of Sema 1a expression (Figure 2D).

We also analyzed the distributions of Sema 1a and Sema 2a in the antero-posterior axis, at the time of sensory axon ingrowth into the CNS, and found they appear uniform (Figure S2A and S2B).

To confirm that Sema 2a and Sema 1a act as the ligands for Plex B and Plex A in our experiments, we tested the *sema 2a* and *sema 1a* dependence of the Plex B and Plex A overexpression phenotypes in sensory neurons.

**Figure 3:**
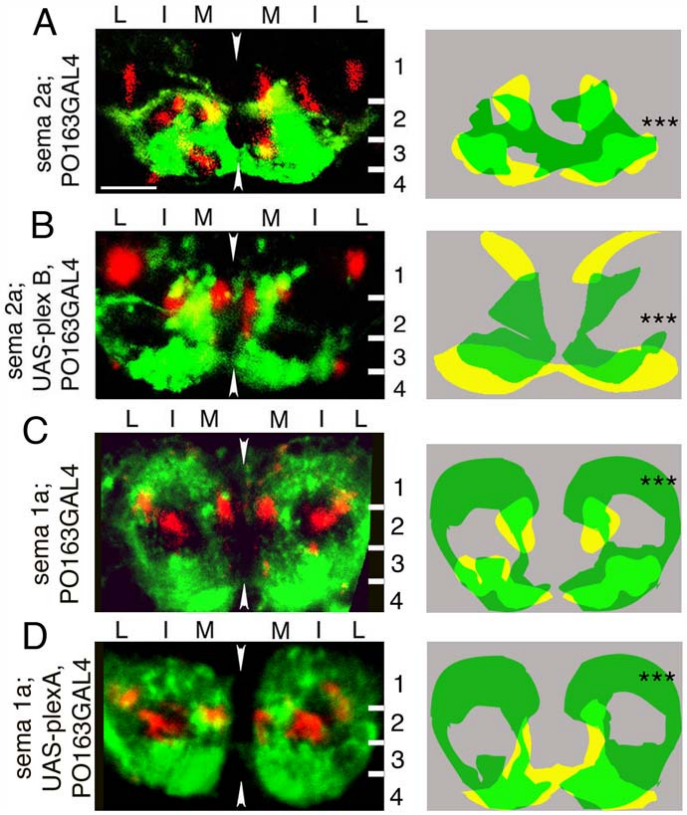
*sema 2a* and *sema 1a* mutations suppress the phenotypes of Plex B and Plex A overexpression in sensory neurons. (A–D) Representative images of sensory terminals labelled with *PO163GAL4, UAS-n-syb-GFP* (green) with respect to Fas II tracts (red) in 21-h embryos (left) and diagrams showing patterns of sensory terminals in different genotypes superimposed (right). In all cases images show projections of a confocal z series of transverse sections through A7. Dorsal is up. Arrowheads show midline. White lines, layer boundaries. Numbers indicate layers: M, medial; I, intermediate; L, lateral domains. Scale bar: 10 µm. (A) In *sema 2a^03021^* mutant embryos, sensory terminals aberrantly invade neuropile layer 2 and to a lesser extent layer 1. Right: Diagram showing the pattern of sensory terminals in *sema 2a^03021^* mutant (green) and (yellow) embryos, superimposed. Quantification of SA in layer 2 in *sema 2a^03021^* mutant embryos reveals a significant increase (***, *p* = 5×10^−5^; Student's *t*-test; average *SA*_2/h_ = 0.1; SD = 0.05; *n* = 24 hemisegments) with respect to wild type (average *SA*_2/h_ = 0.06; SD = 0.02; *n* = 32 hemisegments). (B) Expressing Plex B in sensory neurons in a *sema 2a^03021^* mutant background fails to exclude sensory terminals from neuropile layer 2. Right: Diagram showing the patterns of Plex B expressing sensory terminals in *sema 2a^03021^* (green) mutant and wild-type (yellow) backgrounds, superimposed. Quantification of SA in layer 2 reveals a significant increase (***, *p* = 10×10^−12^; Student's *t*-test; average *SA*_2/h_ = 0.06 and SD = 0.03, *n* = 31 hemisegments), with respect to Plex B expression in wild-type background (average *SA*_2/h_ = 0.003; SD = 0.006, *n* = 24 hemisegments). (C) In *sema 1a^P1^* mutant embryos, sensory terminals aberrantly invade neuropile layer 1 and to a lesser extent layers 2 and 3. This results in an large increase in the surface area occupied by sensory neuron terminals in layers 1 and 3. Right: Diagram showing the patterns of sensory terminals in *sema 1a^P1^* mutant (green) and (yellow) embryos, superimposed. Quantification of *SA*_1+3+4/h_ reveals a significant increase (***, *p* = 5×10^−15^; Student's *t*-test) in *sema 1a^P1^* mutants (average *SA*_1+3+4/h_ = 0.6 and SD = 0.09, *n* = 23 hemisegments), compared to wild type (average *SA*_1+3+4/h_ = 0.3; SD = 0.05, *n* = 32 hemisegments). (D) Expressing Plex A in sensory neurons in a *sema 1a^P1^* background fails to exclude sensory terminals from neuropile layers 1 and 3. Right: Diagram showing the patterns of sensory terminals that express Plex A in *sema 1a^P1^* mutant (green) and wild-type (yellow) backgrounds, superimposed. Quantification of *SA*_1+3+4/h_ reveals a significant increase (***, *p* = 2×10^−25^; Student's *t*-test) in *sema 1a^P1^* mutant (average *SA*_1+3+4/h_ = 0.5 and SD = 0.09, *n* = 30 hemisegments), compared to wild-type (average *SA*_1+3+4/h_ = 0.1; SD = 0.04; *n* = 38) backgrounds.

We analysed patterns of sensory terminals in *sema 2a^03021^* loss of function embryos [25] and in embryos in which *plex B* was overexpressed in sensory neurons in a *sema 2a^03021^* background. In *sema 2a^03021^* embryos we find ectopic sensory terminals in layer 2 (Figure 3A). Overexpression of Plex B in sensory neurons in a *sema 2a^03021^* background fails to exclude sensory terminals from central and dorsal neuropile (compare Figures 2C and 3B). The pattern of sensory terminals in these embryos is similar to their pattern in *sema 2a^03021^* mutants (compare Figure 3A and 3B). We conclude that Sema 2a is the functional ligand for Plex B in this system.

We recombined the *UAS-plex A-HA* [24] transgene with the *sema 1a^P1^* [26] mutation to express Plex A in a *sema 1a* mutant background. We analysed patterns of sensory terminals in *sema 1a^P1^* mutant embryos and in embryos in which Plex A was overexpressed in sensory neurons in a *sema 1a^P1^* background. In *sema 1a^P1^* embryos, we found ectopic sensory terminals in layers 1 and 3 (Figure 3C). Overexpression of Plex A in sensory neurons in a *sema 1a^P1^* background failed to exclude them from layers 1 and 3 (compare Figures 2D and 3D). The pattern of sensory terminals in these embryos is strikingly similar to their pattern in *sema 1a^P1^* mutants (compare Figure 3C and 3D). We conclude that Sema 1a is the functional ligand for Plex A in this system.

We were able to identify potential cellular sources of the transmembrane Semaphorin Sema 1a by looking for neuronal populations that project to layers 1 and 3 (Figure S3). One such population are the motorneurons, most of which project dendrites to layer 1 (Figures 1 and S3A). Using the *OK371-GAL4* we targeted the expression of the cell death gene *reaper* and of the CD8GFP reporter (*OK371-GAL4*, *UASCD8GFP;UAS-reaper*) to the motor neurons [27]. This resulted in the death of most motor neurons by the early first instar larval stage (as judged both by the onset of larval paralysis and by the loss of GFP signal) (Figure S3C). Immunofluorescence visualisation of Sema 1a shows a significant reduction in Sema 1a levels in layer 1 in animals that lack motor neurons, compared to animals with intact motor neurons (Figure S3B, S3D, and S3E). We conclude that the motorneuron dendrites are likely to be a source of Sema 1a in the dorsal neuropile. Another cell population that projects to layer 1, as well as to layer 3, are the GABAergic interneurons (Figure S3F). We used *GADGAL4* [28],[29] to visualise and kill both the motor neurons and the GABAergic interneurons and found that this resulted in nearly complete loss of Sema 1a staining from both layers 1 and 3 (Figure S3G, *n* = 10 embryos). We conclude that the GABAergic interneurons are likely to be a significant source of Sema 1a in layer 1 and also in layer 3.

We have so far been unable to identify cellular populations that project exclusively to layer 2, but since the expression is continuous across the midline (see Figure S2A), at least some midline cells could be involved. One possibility is that the recently described extensions of midline glial cells, the gliopodia [30], might provide a vehicle by which high levels of Sema 2a are deployed across the developing neuropile. Interestingly these extensions of the glial cells have a limited life span, becoming much reduced late in embryogenesis and we find that Sema 2a expression also declines in these late stages. The VNC of embryos that lack midline glial cells (for example in *single minded* mutants; [31]) are too fragile and disorganized to allow analysis of levels of Sema 2a along the dorso-ventral axis. Instead we restored Sema 2a expression in the midline glial cells using the *single mindedGAL4* line [31], in an otherwise *sema 2a* mutant background (*sema 2a*, *UAS-sema 2a*;*single-mindedGAL4*) (see Figure S4A–S4C for details of these experiments). We were able to restore Sema 2a expression in the neuropile (Figure S4B), in layers 1, 2, and 3 (Figure S4C), but in a pattern that appeared broader than the endogenous stripe in layer 2. Thus a source of the Sema 2a gradients could potentially be a subset of the midline cells, although we cannot exclude the possibility that some other cells are the endogenous source of this cue in the CNS.

### Ventrally Projecting Sensory Neurons Terminate in Regions of Low Sema 1a and Low Sema 2a Expression Levels

To investigate the role of the Sema/Plexin system in determining the position at which axons terminate within the layered structure of the neuropile we decided to focus our experiments on a single class of sensory cells with well defined terminal branches, the nociceptive class IV md neurons. Class IV md neurons can be identified with *ppkEGFP* [9],[12], which labels one ISN and two SN neurons in each hemisegment. The axons of these cells terminate medially in the ventral-most part of the neuropile, layer 4 (Figure 1C), where they branch asymmetrically in the antero-posterior axis (Figure S7A).

**Figure 4:**
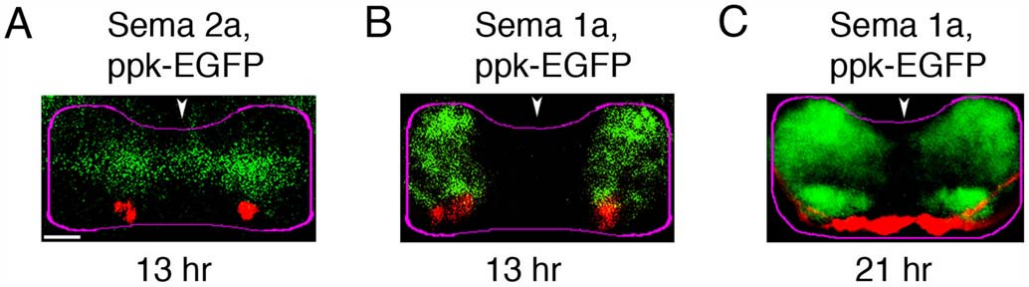
Class IV md neurons terminate in a neuropile region with low levels of Sema 1a and Sema 2a. Immunofluorescence visualisation of Sema 2a (A) and Sema 1a (B and C)) (green) with respect to ppk-EGFP (red), in the CNS at 13 (A and B) or 21 (C)-h AEL. Images show projections of a confocal z series of transverse sections through A7. (A) Class IV axon terminals are in a region of low Sema 2a levels. (B and C) Class IV axons, grow to ventral-most neuropile, where Sema 1a levels are lowest. Class IV terminals are just beneath regions of higher Sema1a expression levels in layer 3 at both 13 (B) and 21 (C)-h AEL. Note that neither Sema 1a nor Sema 2a is expressed at high levels in most lateral regions of neuropile, thus providing a potential corridor for axons to grow from their entry points to more ventral neuropile regions. Dorsal up. Arrowheads show midline. Magenta lines, neuropile outlines. Scale bar: 5 µm.

By examining the location of these *ppkEGFP*-expressing axons with respect to Sema expression we confirmed that at 13-h AEL these axons terminate in a region of low Sema 2a (Figure 4A) and just below regions of high Sema 1a expression levels (Figure 4B). At 21-h AEL the class IV terminals remain in a region with low Sema 1a expression (Figure 4C).

### *sema 1a* and *sema 2a* Are Required to Exclude Ventrally Projecting Class IV md Neurons from Dorsal and Central Neuropile

**Figure 5:**
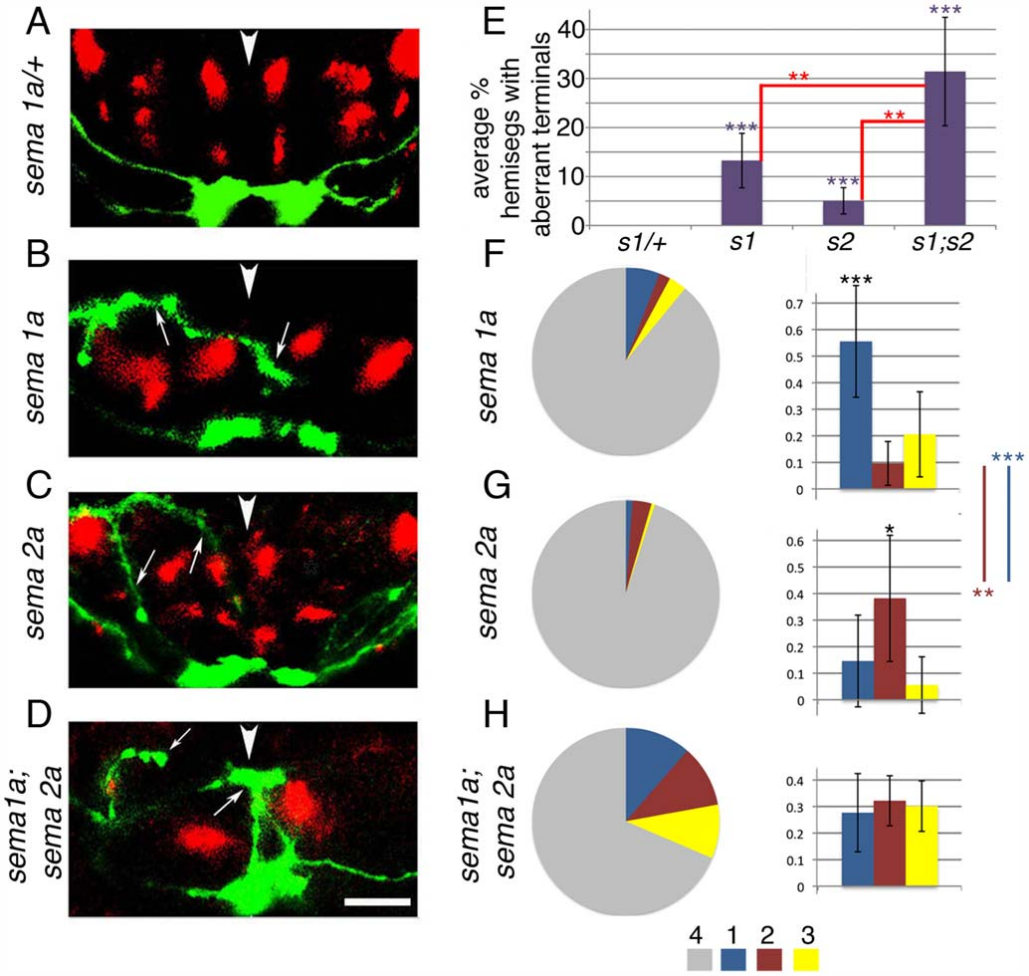
*sema 1a* and *sema 2a* are required to exclude Class IV axons from dorsal and central neuropile. (A–D) Projections of class IV axons labelled with *ppkEGFP* (green) and Fas II tracts (red) in *sema 1a^P1^/+* (A), *sema 1a^P1^* (B), *sema 2a^03021^* (C), and *sema 1a^P1^*, *sema 2a^03021^* double mutant (D) 21-h embryos. Images show projections of confocal z series of transverse sections through an abdominal segment. (A) Projections of Class IV axons in *sema 1a^P1^/+; ppkEGFP* embryos. (B) In *sema 1a^P1^; ppkEGFP* embryos class IV axons project aberrantly and terminate in dorsal and central regions of neuropile (arrows). Fas II tracts are also severely affected. (C) In *sema 2a^03021^; ppkEGFP* embryos class IV axons project aberrantly to dorsal and central neuropile (arrows). The position of Fas II tracts is also affected. (D) Class IV projections have a significantly stronger phenotype in *sema 2a^03021^, sema 1a^P1^; ppkEGFP* embryos than in single mutants and often terminate in dorsal neuropile. Two class IV axons in the left hemisegment have initially entered the CNS centrally and then turned and terminated in dorsal neuropile (arrow). The disruption of Fas II labelled tracts is also stronger in double mutants. Dorsal up. Arrowheads show midline. Scale bar: 8 µm. (E–H) Quantification of aberrant termination phenotypes. For our statistical analysis of class IV termination phenotypes we counted as “aberrant” only those hemisegments with terminals in layers 1, 2, or 3 (Figure S5A and S5B). We did not count as “aberrant” those axons that grow aberrantly through layers 1, 2, or 3, without terminating there (*aberrant growth with normal termination*) (see Figure S5C). (E) Chart shows average percentage of hemisegments with aberrant terminals (Hat) in layers 1, 2, and 3 per embryo (per 14 hemisegments) [Hat(1+2+3) = (*n* hemisegments with terminals in 1, 2, and 3/14)×100] in *sema 1a^P1^/+* (s1/+), *sema 1a^P1^* (s1), *sema 2a^02021^* (s2), and *sema 1a^P1^*, *sema 2a^03021^* double mutant (s1, s2) 21-h embryos. Hat(1+2+3) is significantly higher in *sema 1a^P1^* (purple ***, *p* = 1×10^−5^; Student's *t*-test; average Hat(1+2+3) = 13%; *n* = 21 embryos, 294 hemisegments), *sema 2a^03021^* (purple ***, *p* = 6×10^−5^; Student's *t*-test; average Hat(1+2+3) = 5%; *n* = 24 embryos, 336 hemisegments) and *sema 1a^P1^*, *sema 2a^03021^* double mutant (purple ***, *p* = 7×10^−4^; Student's *t*-test; average Hat(1+2+3) = 31.3%; *n* = 10 embryos, 140 hemisegments) than in *sema 1a^P1^*/+ controls (average Hat(1+2+3) = 0%; *n* = 30 embryos, 420 hemisegments). Hat(1+2+3) is also significantly higher in *sema 1a^P1^*, *sema 2a^03021^* double mutants, than in either *sema 1a^P1^* (red **, *p* = 0.01) or *sema 2a^03021^* (red **, *p* = 0.002) single mutants. (F–H) Pie charts (left) show proportion of hemisegments with class IV axon terminals in layers 1, 2, 3, and 4 in the different mutant backgrounds (out of the total number of hemisegments analysed and pooled from different embryos). Bar charts (right) show the average (per embryo) relative proportion of hemisegments with aberrant terminal in each layer, in the different mutant backgrounds. Blue, Hat(1)/Hat(1+2+3); red, Hat(2)/Hat(1+2+3); yellow, Hat(3)/Hat(1+2+3). (F) In *sema 1a* mutants 87% of axons terminate in layer 4, and 13% terminate aberrantly (*n* = 294). 7% of axons terminate in layer 1, 2% in layer 2, and 4% in layer 3. Right: In *sema 1a* mutants there is a significantly higher proportion of aberrant hemisegments with terminals in layer 1 than in layer 2 (black ***, *p* = 4×10^−5^). Comparison with *sema 2a* mutants reveals a significantly higher proportion of aberrant hemisegments with terminals in layer 1 (blue line and blue ***, *p* = 5×10^−4^). (G) In *sema 2a* mutants 95% of axons terminate in layer 4, and 5% terminate aberrantly (*n* = 336). 3% of axons terminate in layer 2 and 2% in layer 3. Right: In *sema 2a* mutants there is a significantly higher proportion of aberrant hemisegments with terminals in layer 2 than in layer 1 (black *, *p* = 0.027). A comparison with *sema 1a* mutants reveals a significantly higher proportion of aberrant hemisegments with terminals in layer 2 than in *sema 1a* mutants (red line and red **, *p* = 0.005). (H) In *sema 1a, sema 2a* double mutants 69% of axons terminate in their wild-type layer 4, and 31% terminate aberrantly (*n* = 140). 11% of axons terminate in layer 1, 11% in layer 2, and 9% in layer 3.

We now asked whether *sema 1a* and *sema 2a* are required to confine class IV projections to layer 4. In embryos mutant for *sema 1a^P1^* [26], *sema 2a^03021^* [25], and in *sema 1a^P1^*, *sema 2a^03021^* double mutants, the class IV axons have aberrant patterns of termination and/or growth in the dorso-ventral axis (compare Figure 5A with 5B, 5C and 5D; see also Figure S6 for details of the effects of these mutations on the dorso-ventral position of Fas II tracts). We make a distinction between growth and termination phenotypes of class IV axons (For details of this distinction and examples of different kinds of growth and termination phenotypes see Figure S5).

We found a significant increase in the percentage of hemisegments with aberrant terminals in *sema 1a^P1^*, *sema 2a^03021^*, and *sema 1a^P1^*, *sema 2a^03021^* double mutants with respect to *sema 1a^P1^*/+ controls (Figure 5A–5H). Moreover, the percentage of hemisegments with aberrant termination in *sema 1a^P1^*, *sema 2a^03021^* double mutants, was significantly higher than in either *sema 1a^P1^* or *sema 2a^0302^* single mutants (Figure 5E).

We found that in *sema 1a^P1^* mutants aberrant class IV axons tend to terminate in layer 1 more often than in layer 2 (Figure 5F). Conversely, in *2a^03021^* mutants, we found that aberrant class IV axons tend to terminate in layer 2 more often than in layer 1 (Figure 5G). In *sema 1a^P1^*, *sema 2a^03021^* double mutants (Figure 5H) class IV axons terminate with roughly equal probability in layers 1, 2, or 3. Sema 1a appears to play a more important role in preventing termination in layer 1, followed by layer 3, and a minor role in preventing termination in layer 2. Sema 2a appears to play a more important role in preventing termination in layer 2, and a minor role in preventing termination in layers 1 and 3. Our results suggest that Sema 1a and Sema 2a are instructive for termination of class IV axons along the dorso-ventral axis.

We also assessed the potential roles of Sema 1a and Sema 2a in controlling the termination of class IV axons in the antero-posterior axis by analysing their projections in a top-down view of the neuropile in wild type and in *sema 1a*, *sema 2a* double mutants (Figure S7). We chose the *sema 1a*, *sema 2a* double mutant for this analysis, because it exhibited the strongest phenotypes in the dorso-ventral axis. Wild-type class IV axons grow asymmetrically, within their normal ventral and medial termination domain, forming thicker terminal in the anterior than in the posterior portion of the segment (Figure S7A). We did not observe a significant loss of this asymmetry in the *sema 1a, sema 2a* double mutant compared to wild type (Figure S7B and S7C). In top down view, class IV terminals do appear disorganized compared to wild type, but we assume this disorganization is a consequence of the major defects in growth and termination in the dorsoventral axis. Thus Sema 1a and Sema 2a do not appear to play a major role in confining class IV terminals to the anterior portion of the segment. Consistent with this idea is also our finding that the distributions of Sema 1a and Sema 2a appear uniform in the antero-posterior axis, at the time of sensory axon ingrowth into the CNS (Figure S2A and S2B).

### *sema 1a* Is Required Non-Cell-Autonomously to Exclude Class IV Axons from Dorsal Neuropile

In some cases membrane-bound Sema 1a acts as a receptor [18],[32]. Thus, rather than a requirement to act as a cue, the class IV mutant phenotypes could reflect a cell-autonomous requirement for Sema 1a in the sensory neurons themselves. To resolve this, we performed two kinds of rescue experiments.

**Figure 6:**
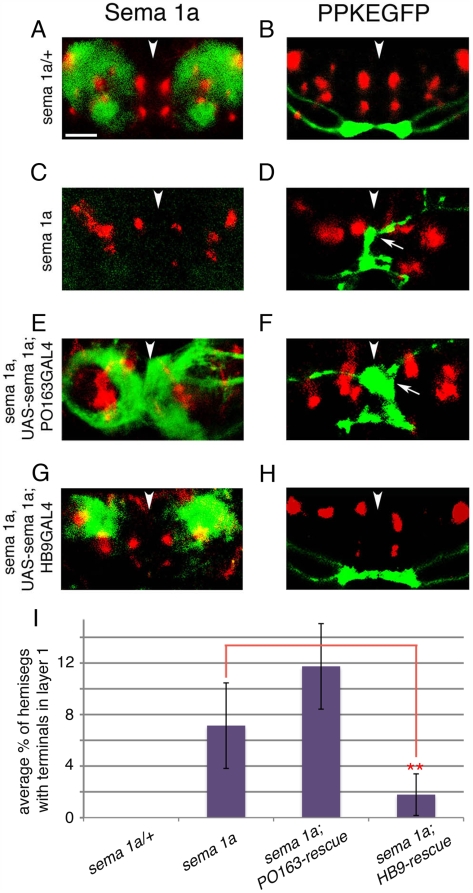
*sema 1a* is required non-cell autonomously in dorsal neuropile for the ventral projection of Class IV axons. (A, C, E, and G) Immunofluorescence visualisation of Sema 1a (green) with respect to Fas II tracts (red), in *sema 1a/+* control (A), *sema 1a^PL^* mutant (C), *sema 1a^PL^UAS-sema 1a;PO163GAL4* (E), and *sema 1a^PL^UAS-sema 1a;HB9GAL4* (G) embryos (21-h old). Images show projections of a confocal z series of transverse sections through A7. (A) Normal pattern of Sema 1a expression in 21-h-old embryos. (C) Sema 1a is absent in *sema 1a^PL^* embryos. (E) Sema 1a is expressed selectively in sensory neuron axons in the *sema 1a^PL^UAS-sema 1a;PO163GAL4* embryos. Note aberrant entry of many sensory axons into dorsal neuropile in the absence of Sema 1a. (G) Sema 1a is expressed selectively in motor neuron dendrites in dorsal neuropile, in *sema 1a^PL^, UAS-sema-1a; HB9GAL4* embryos. (B, D, F, and H) Projections of class IV axons (green) and Fas II tracts (red) in *sema 1a/+* control (B), *sema 1a^PL^* mutant (D), *sema 1a^PL^UAS-sema 1a;PO163GAL4* (F), and *sema 1a^PL^UAS-sema 1a;HB9GAL4* (H) embryos (21-h old). Images show projections of confocal z series of transverse sections through an abdominal segments. (B) Normal class IV md projections. (D) In *sema 1a^PL^;ppkEGFP* embryos class IV axons project aberrantly and terminate in dorsal and central regions of the neuropile (arrow). (F) Restoration of Sema 1a expression in sensory neurons alone in *sema 1a^PL^, UAS-sema-1a; PO163GAL4, ppkEGFP* embryos does not prevent the aberrant dorsal projection (arrow) of class IV neurons. (H) Expression of Sema 1a in motor neuron dendrites in *sema 1a^PL^, UAS-sema-1a; HB9GAL4, ppkEGFP* embryos reduces the aberrant dorsal projection of class IV terminals. Dorsal up. Arrowheads show midline. Scale bar: 8 µm. (I) Quantification of termination phenotypes in the different genetic backgrounds. Graph shows average percentage of hemisegments per embryo (per 14 abdominal hemisegments) with aberrant class IV terminals in layer 1 [Hat(1) = (*n* hemisegments with terminals in 1/14)×100], in *sema 1a^PL^*/+, *sema 1a^PL^* mutant, *sema 1a^PL^UAS-sema 1a;PO163GAL4* and *sema 1a^PL^UAS-sema 1a;HB9GAL4* embryos. Hat(1) is significantly higher in *sema 1a^PL^* (*p* = 5×10^−5^; Student's *t*-test; average Hat(1) = 7%; *n* = 21 embryos, 294 hemisegments) and in *sema 1a^PL^*, *UAS-sema 1a; PO163-GAL4* embryos (*p* = 8×10^−6^; Student's *t*-test; average Hat(1) = 14 embryos, 196 hemisegments), than in *sema 1a^PL^*/+ controls (average Hat(1) = 0%, *n* = 30 embryos, 420 hemisegments). In *sema 1a^PL^*, *UAS-sema 1a; PO163-GAL4* embryos there is no reduction in the percentage of hemisegments with terminals in layer 1 compared to *sema 1a^PL^* mutant embryos. *sema 1a^PL^*, *UAS-sema 1a; HB9-GAL4* rescue embryos show a significant reduction in hemisegments with terminals in layer 1 (red **, *p* = 0.004; Student's *t*-test; average Hat(1) = 1.8%, *n* = 12 embryos, 168 hemisegments), compared to *sema 1a^PL^* mutant embryos (average Hat(1) = 7%).

First, we restored *sema 1a* expression to sensory neurons in *sema 1a^P1^*mutant embryos using *PO163GAL4*. Antibody labelling confirms that Sema 1a is successfully targeted to embryonic sensory terminals using this driver (compare Figure 6A, 6C, and 6E) and shows that in the mutant a large fraction of Sema 1a-expressing sensory neurons aberrantly project to the dorsal part of the neuropile (Figure 6E). We then analysed specifically the projections of class IV neurons in embryos where *sema 1a* expression had been restored to sensory neurons in the *sema 1a^P1^* mutant background (compare Figure 6B, 6D, and 6F). There was no rescue of the dorsal termination phenotype of class IV axons in these embryos. Quantification revealed no significant reduction in dorsal termination of class IV axons, compared to *sema 1a^P1^* mutants (Figure 6I). Thus, *sema 1a* is not required in class IV neurons themselves to exclude their terminals from dorsal neuropile.

In a second set of experiments, we selectively restored Sema 1a to dorsal neuropile in an otherwise *sema 1a^P1^* mutant background, by using *HB9GAL4* to drive its expression in a subset of motor neurons [33]. We used the *HB9GAL4* line for this rescue experiment, because it is expressed before sensory neurons grow into the neuropile, unlike *GADGAL4* or *OK371GAL4*, which are expressed later. We confirmed that Sema 1a is selectively present in dorsal neuropile in these experiments (compare Figure 6A, 6C, and 6G), and we observed a significant reduction in the dorsal termination of class IV axons compared to *sema 1a^P1^* mutants (Figure 6H and 6I). Thus in an otherwise mutant background, the mutant phenotype of class IV axons can be partially rescued by expressing *sema1a* dorsally in the dendrites of motor neurons.

We also asked whether restoration of Sema 2a expression in the midline glial cells using the *single mindedGAL4* line in an otherwise *sema 2a* mutant background (*sema 2a, UAS-sema 2a;single-mindedGAL4, ppkeGFP*) rescues the *sema 2a* mutant phenotype of class IV axons (see Figure S4 for details of these experiments). We observed a significant reduction in the aberrant termination of class IV axons in layer 2 compared to *sema 2a* mutant embryos (Figure S4D and S4E).

### *plex A* and *plex B* Are Both Required to Exclude Class IV Terminals from Regions with High Sema 1a and Sema 2a Expression Levels

The experiments we describe suggest that both Sema 1a and Sema 2a are required as cues to confine class IV sensory axons to ventral neuropile. We also find that expressing either *plex A* or *plex B* in sensory neurons is sufficient to shift their terminals away from regions with high levels of Sema 1a and 2a. Thus, a combination of Plexins could be required in ventrally projecting sensory neurons to exclude them from dorsal and central neuropile.

By in situ hybridization we confirmed previous reports [20] that ch, dbd, and class I–IV md neurons express *plex B* at the time that sensory axons grow into and terminate in the VNC (Figure S8D). By double labelling with anti-Plex A and anti-horseradish peroxidase we confirmed that Plex A is expressed in sensory neuron cell bodies at 13-h AEL (Figure S8A), and by double labelling with anti-Plex A and anti-GFP showed that Plex A is strongly expressed in the *ppk*-expressing class IV neurons (Figure S8B). Unfortunately, none of these experiments allows us to draw quantitative conclusions about levels of expression in different cells. High background levels also prevented a reliable analysis of Plex A expression along the dorso-ventral axis of the CNS. However antibody labelling against Plex A does reveal expression in the neuropile at 13-h AEL (Figure S8C).

To show whether both Plexins are required to exclude the ventrally projecting sensory neurons from central and/or dorsal neuropile, we analysed the projection pattern of class IV axons in *plex A* and *B* mutants.

**Figure 7:**
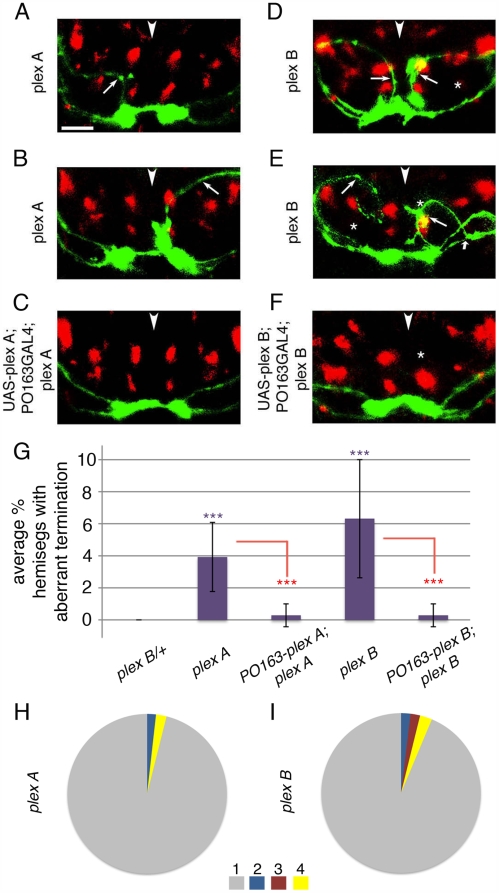
Plex A and Plex B are required in sensory neurons for the ventral termination of Class IV axons. Projections of class IV axons (green) and Fas II tracts (red) in *plex A* (A and B), *plex B* (D and E) mutant, and *UAS-PlexA;PO163GAL4;plexA* (C), and *UAS PlexB;PO163GAL4;plexB* (F) rescue embryos (21 h). Images show projections of confocal z series of transverse sections through an abdominal segment. (A and B) In *ppkEGFP; plex A* embryos, class IV axons project aberrantly through dorsal and central regions (arrows) of neuropile. Fas II tracts appear wild type. (C) Rescue of Plex A expression in sensory neurons alone in *UAS-plexA;PO163GAL4,ppkEGFP;plexA* embryos is sufficient to prevent aberrant projection of class IV axons to dorsal and central regions of the neuropile. (D and E) In *ppkEGFP; plexB* embryos class IV axons are severely affected. They grow aberrantly through and often terminate in dorsal and central neuropile (thin arrows). Sometimes they project initially to ventral neuropile (thick arrow in E), then turn dorsally and terminate in dorsal neuropile. The dorsoventral and mediolateral positions of Fas II tracts are also altered in these mutants (white *, wild-type Fas II tract positions). Severity of Fas II phenotype does not correlate with severity of sensory axon phenotype. (F) Rescue of Plex B expression in sensory neurons alone, in *UAS-plexB;PO163GAL4,ppkEGFP;plexB* embryos prevents aberrant projection of class IV terminals to dorsal and central neuropile. Fas II tracts continue to exhibit the mutant phenotype in these embryos (x denotes wild-type position of M1 tract). Dorsal up. Arrowheads show midline. Scale bar: 8 µm. (G–I) Quantification of termination phenotypes. (G) Chart shows average percentage of hemisegments with aberrant terminals (Hat) in layers 1, 2 and 3 per embryo (per 14 hemisegments) [Hat(1+2+3) = (*n* hemisegments with terminals in 1, 2, and 3/14)×100] in *plex B/+* control; *plex A* mutants and *UAS-plex A;PO163GAL4,ppkEGFP;plex A* rescue; *plex B* mutants and *UAS-plex B; PO163GAL4,ppkEGFP; plex B* rescue embryos. A significantly higher percentage of hemisegments with aberrant class IV terminals in layers 1, 2, or 3 is observed in both *plex A* mutants (purple ***, *p* = 3×10^−4^; Student's *t*-test; average Hat(1+2+3) = 4%; *n* = 20 embryos, 280 hemisegments) and *plex B* mutants (purple ***, *p* = 10×10^−5^; Student's *t*-test; average Hat(1+2+3) = 6%; *n* = 26 embryos, 364 hemisegments), compared to plex B/+ control embryos (average Hat(1+2+3) = 0%; *n* = 25 embryos, 350 hemisegments). The percentage of hemisegments with aberrant class IV terminals in *plex A-rescue* is significantly lower (red ***, *p* = 8×10^−4^; Student's *t*-test; *n* = 25 embryos, 350 hemisegments) compared to *plex A* mutants and not significantly different from *plex B/+* controls. Likewise, the percentage of hemisegments with aberrant class IV terminals in *plex B-rescue* is significantly lower (red ***, *p* = 2×10^−4^; Student's *t*-test; *n* = 25 embryos, 350 hemisegments) compared to *plex B* mutants and not significantly different from *plex B/+* controls. (H and I) Pie charts show proportion of hemisegments with class IV axon terminals in layers 1, 2, 3, and 4 (out of the total number of hemisegments analysed pooled from different embryos), in *plex A* embryos (*n* = 280) (H) and *plex B* embryos (*n* = 364) (I). In *plex A* mutants 96% of class IV axons terminate in layer 4, whereas 4% aberrantly terminate in layers 1 (2%), or 3 (2%). In *plex B* mutants 93.7% of class IV axons terminate in layer 4, whereas 6.3% aberrantly terminate in layers 1 (1.9%), 2 (1.9%), or 3 (2.5%).

In *plex A^Df(4)C3^* mutants, class IV axons project aberrantly to central and/or dorsal neuropile (Figure 7A and 7B). Quantification reveals significantly more terminals in dorsal and central neuropile, compared to wild type (Figure 7G).

In *plex B^KG00878^* mutants class IV axons also project to dorsal or central neuropile (Figures 7D and 7E). Quantification reveals significantly more terminals in dorsal and central neuropile compared to wild type (Figure 7G).

We also quantified the proportion of terminals in each of the different layers of the neuropile (Figure 7H and 7I). We found that in *plex B* mutants Class IV axons terminate with roughly equal probability in layers 1, 2, or 3. This suggests that Plex B may normally have a role in preventing termination in layers with high levels of Sema 2a or Sema 1a and may therefore be a functional receptor for both ligands. We also observed that embryos transheterozygous for *plex B* and either *sema 1a* (*sema 1a/+; plex B/+*), *sema 2a* (*sema 2a/+; plex B/+*), or *plex A* (*plex B/plex A*) all exhibit class IV termination phenotypes, indicating a genetic interaction between these mutations (unpublished data). To further test whether Plex B functions to prevent termination in regions with high Sema 1a levels we analysed the patterns of sensory terminals that overexpress Plex B in a *sema 1a* mutant (Figure S9). In these embryos we observed a striking expansion of sensory terminals into the intermediate region of layer 1 that normally contains highest Sema 1a levels within this layer (compare Figure S9A and S9B). In a wild-type background Plex B-overexpressing sensory terminals remain confined in the most medial portion of layer 1, even though they become excluded from layer 2 (Figures 2C, S9A). A similar expansion is also observed in *plex A^Df(4)C3^* mutants, indicating that Plex A function is required to prevent termination in regions with highest levels of Sema 1a (Figure S9C). Interestingly, we found that in the absence of *plex A*, Plex B overexpression in sensory neurons is sufficient to prevent their expansion into regions with highest Sema 1a levels (in *PO163GAL4, UAS-plex B; plex A^Df(4)C3^* embryos) (Figure S9D).

### Rescuing *plex A* and *plex B* in Sensory Neurons Alone Is Sufficient to Prevent the Aberrant Projection of Class IV Neurons to Dorsal and Central Neuropile

To exclude (a) the possibility that *plex A* and *B* are required in the central targets of sensory neurons, in which case the mutant phenotypes might be a result of aberrations in normal target directed growth and (b) the possibility that Plex A and B are acting as guidance cues we restored their expression selectively to sensory neurons using the *P0163 GAL4* driver. Restoration of Plex B expression selectively in sensory neurons in a *plex B^KG00878^* mutant background, rescues the phenotype of class IV md neurons (Figure 7E). Quantification reveals significantly fewer aberrant class IV terminals in *plex B-rescue* embryos as compared to *plex B* mutants (Figure 7G). The Fas II tracts continued to exhibit mutant phenotypes in these experiments, as would be expected if the rest of the neuropile, other than the sensory neurons, remained mutant (Figure S10).

Likewise, restoration of Plex A expression in sensory neurons in a *plex A^Df(4)C3^* mutant background, rescued the phenotype of class IV md neurons (Figures 7C). Quantification reveals significantly fewer aberrant class IV terminals in *plex A-rescue* embryos compared to *plex A* mutants (Figure 7G). We conclude that both *plex A* and *plex B* are required in sensory neurons for the appropriate targeting of class IV axons to the ventral neuropile.

## Discussion

In the work we report here, we have addressed the general issue of how neuronal termination is regulated within a complex meshwork of differentiating axons and dendrites. Connections are formed within a central neuropile from which cell bodies are excluded. As first noted by Cajal [34], removing cell bodies to the periphery, while multiple connections are formed within a central core, maximises the economy with which the network is wired together. As a consequence of this organisation the processes of neurons of all kinds—motor, sensory, and interneurons—grow into a common volume of the nervous system within which connections will be formed. This growth is patterned and consistent, so that the forming network is partitioned into different domains within which limited subsets of neurons terminate and form characteristic arborisations. In the VNC of *Drosophila* embryo, for example, motor neurons place their dendrites in the most dorsal domain of the neuropile where they arborise to form a myotopic map that represents centrally the distribution of innervated muscles in the periphery [35]. While these dendritic maps are forming dorsally, the axons of sensory neurons are growing into the same neuropile and terminating in other characteristic and consistent domains. Here too, each modality is targeted to a particular volume of the neuropile where the terminals form a characteristic pattern of arborisations [7].

In a system in which neither the axonal nor dendritic terminals are constrained by the cell bodies or by the position of entry of the main dendrite or axon trunk into the neuropile, we envisage that connectivity develops in stages with an initial phase in which growing axons and dendrites are both delivered to appropriate volumes of the neuropile, followed by a phase of targeted connection between appropriate pre- and postsynaptic partners. A pattern of growth of this kind would resemble that seen in the developing olfactory system of the adult fly where coarse targeting to particular regions of the antennal lobe is followed by precise recognition and matching between axons and dendrites [36],[37]. Alternatively it may be that the mechanisms that pattern the growth of fibres representing a single modality such as olfaction are different from those required to organise the distribution of terminals within a highly heterogeneous network such as that seen in the VNC. In our view the initial delivery and restriction of fibres to particular subvolumes of the VNC neuropile is likely to be by individual growth responses to generalised systems of cues that operate to pattern the network as it develops. In a previous paper we were able to demonstrate the operation of one such cue, Slit, which, acting through its Robo receptors dictates the different positions in the mediolateral axis at which specific sensory axons will terminate and arborise. Here we have shown that membrane bound and secreted Semas acting through their receptors the Plexins restrict growing axons and their terminals to particular dorso-ventral layers of the forming neuropile.

### Evidence for Sema/Plexin Signalling Acting in the Dorso-Ventral Axis of the Neuropile

Our initial approach of using a misexpression screen targeted to all sensory axons was sufficient to reveal the existence of the Semas as putative cues in the dorso-ventral axis by showing that there were generalised redistributions of sensory endings in this axis when the cells concerned were forced to express either of the two Sema receptors, Plex A or Plex B. These shifts were readily detectable when the nervous system was viewed in a plane at right angles to the neuraxis.

Viewing the nervous system in this plane also reveals the largely complementary patterns of expression for the two Semas. The membrane bound Sema 1a is distributed in an alternating pattern across the neuropile with high levels in both layers 1 and 3. The secreted Sema, Sema 2a, on the other hand, is expressed at high levels in a central strip that extends across the midline and in gradients that decline ventrally and dorsally orthogonal to the Slit gradient (Figure 2). A gradient of Sema 2a has also been described in the embryonic limb of the grasshopper [38]. There it contributes to the polarized growth of pioneer sensory axons away from the region of highest Sema 2a expression at the tip.

In the developing *Drosophila* embryo selective overexpression of the putative receptors for Sema 1a and Sema 2a in sensory neurons acts in a predictable fashion to exclude sensory axons and terminals from those regions where the ligands are highly expressed: overexpression of Plex A excludes projections from high levels of Sema 1a expression in layers 1 and 3. Overexpression of Plex B shifts sensory terminals further away from the central layer of the neuropile. These findings suggest that Sema 2a and Sema 1a provide guidance cues to the growth cones of sensory neurons that express Plex A and Plex B. It is consistent with this idea that in the absence of Sema 1a, Plex A overexpression in sensory neurons does not exclude their terminals from regions that normally contain high Sema 1a levels. Similarly, in the absence of Sema 2a, Plex B overexpression in sensory neurons does not exclude their terminals from the central layer of the neuropile.

The manipulations of the pattern of sensory terminals in the dorso-ventral axis found with Plexin overexpression are analogous to the manipulations in the medio-lateral axis that are found with Robo3 misexpression. In both dimensions the position at which sensory neurons form their terminals is determined by their expression of receptors for positional cues.

### Sema/Plexin Signalling Guides Termination in the Dorso-Ventral Axis

The most ventrally located sensory terminals, the *ppk*-expressing md neurons are derived from axons that actually enter the nervous system dorsally and grow downwards, skirting alternative neuropile regions before turning inwards to reach their characteristic medial, ventral domain of termination. A consequence of Sema signalling is that these ventrally targeted axons are excluded from more dorsal regions of the neuropile and channelled instead through a limited lateral region where the expression of both proteins is low, so that their inward migration towards the midline is blocked until they reach the most ventral region. In the absence of either of the Semas or their Plexin receptors, *ppk*-expressing axons aberrantly enter and terminate in more dorsal regions of the neuropile. This suggests that the growth cones of these cells are attracted towards to midline (we assume by Netrins) [39] as soon as they enter the CNS, but that entry and termination in the more dorsal region of the neuropile is prevented by high levels of Sema 1a in layer 1.

In vertebrates genetic studies show that proprioceptive axons are excluded from the superficial dorsal horn by Sema 6D/6C signalling mediated by Plex A1. Loss of Plex A1 allows proprioceptive collaterals to invade the superficial dorsal horn although most succeed in projecting through it to their normal more ventral target zones [19]. In an analogous (though not topologically equivalent) fashion, ventrally projecting afferents in *Drosophila* require Sema signalling through Plex A for their proper exclusion from the most dorsal neuropile.

Loss of *plex A* appears to affect class IV terminals less strongly than loss of *sema 1a*. One explanation could be that Plex B might also function as a receptor for Sema 1a in this system. Our observation that in *plex B* mutants class IV axons aberrantly terminate in layers 1, 2, or 3 supports this possibility. We also find that Plex B overexpression in sensory neurons in *plex A* mutant embryos, prevents aberrant expansion of sensory terminals into intermediate portion of layer 1, which contains very high levels of Sema 1a (Figure S9). Such an expansion occurs in both *plex A* and *sema 1a* mutant embryos. High levels of Plex B signalling thus appear to be able to substitute for the absence of Plex A signalling and prevent expansion into regions with high Sema 1a levels. These findings could be explained if Plex B were to function as a lower affinity receptor for Sema 1a, as well as a high affinity receptor for Sema 2a.

Sema 1a and Sema 2a are unlikely to be the only cues that operate in the dorso-ventral axis. The incomplete penetrance of the termination phenotype in the *sema 1a, sema 2a* double mutant suggests that additional factors may operate to control the ventral targeting of class IV axons. There may be long range ventral attractants or local substrate bound attractive cues for these axons in the neuropile. It is also likely that dorsally and centrally located sensory and interneuron terminals, as well as dendrites of motor neurons may require additional signals to exclude them from ventral neuropile. Such signals could be the other Semas. Alternatively, by analogy with the optic tectum, where Wnt signalling drives dorsal projections and Ephrins dictate ventral projections, it is possible that some other signalling system may operate with Semas to confine dorsally projecting neurons to dorsal neuropile [3],[40],[41].

### Type-Specific Repulsion in the VNC

In the fly antennal lobe, during the formation of the olfactory map, Sema 1a expression on the surfaces of antennal olfactory receptor neuron (ORN) axons excludes Plex A expressing maxillary palp ORN axons from inappropriate glomeruli [22],[23].

Our findings suggest that much of the Sema 1a expression in the neuropile of the VNC is on the surfaces of motor neuron dendrites and on the projections of the GABAergic interneurons. Thus, there appear to be two kinds of positional cues in the neuropile. Slit and Sema 2a are examples of secreted and possibly glia-mediated positional cues. Sema 1a on the other hand is presented on membranes of particular neuronal classes (GABAergic interneurons and motorneurons) and is a repellent for the axons of at least one other type of neuron (class IV md neurons). Thus, the presentation of repellent molecules on the surfaces of subsets of neurons can act to exclude specific classes of axons from particular regions of the neuropile.

### Positional Cues Subdivide the Neuropile into Different Termination Domains within Which Connections Form

Theoretical models for gradient-guided axonal growth and targeting during the formation of 2-D neural maps, such as the retinotopic projections, require at least one gradient in each of the two—not necessarily Cartesian—dimensions [42]. These ideas have been borne out by experimental findings. Gradients of attractants and repellents in one dimension have been implicated in providing positional information for terminating sensory axons during the formation of both continuous and discrete neural maps [18],[43],[44]. Furthermore, a recent study has shown that two orthogonal systems of graded cues operate to specify position of termination along each axis of a somatotopic map in the optic tectum.

Our findings address the larger issue of how termination of distinct neuron classes is regulated within a complex meshwork of differentiating axons and dendrites. They suggest that similar mechanisms that are used for the establishment of neural maps, only involving generalized positional cues in each dimension, control targeting of many different classes of neurons to specific termination domains within a complex neuropile.

Although the evidence we provide here suggests that positional cues can specify particular domains for the termination of sensory neurons, we do not suppose that the control of termination and branching by a pervasive system of positional cues would necessarily be sufficient to allow connections to form selectively and specifically between appropriate pre- and postsynaptic partners. What such a system does provide is a framework of signals that could regulate simultaneously the growth of axons and dendrites of many different neurons and induce their termination and branching in appropriate parts of the developing network. Within these restricted regions it is likely that further, localised mechanisms, including competitive interactions, patterns of activity, and target derived cues might all be required to control synaptogenesis and determine the emergence of precise patterns of connectivity within a termination domain.

### Coordinate Positioning of Pre- and Postsynaptic Terminals by the Same Cues?

If the pattern of sensory axon termination within the neuropile is controlled by a system of positional cues, most likely, in three dimensions, it may well be that the location of their postsynaptic dendrites is determined in a similar fashion. If this were the case, the matched expression of receptors for the same system of signals by pre- and postsynaptic neurites would guide them to a common volume as a prelude to the formation of synaptic connections between them. Recent studies that show that developing motor neuron dendrites respond to some of the same cues as terminating sensory axons provide indirect evidence for common systems of positional cues leading to the coordinate targeting of presynaptic axons and postsynaptic dendrites [45],[46]. A direct test of this hypothesis, however, must await the identification of the postsynaptic interneurons with which developing sensory neurons form connections. It will then be possible to make a direct investigation of the molecular mechanisms that control the termination and branching of pre- and postsynaptic endings and thereby lay out a ground plan for connectivity within the developing neuropile.

## Materials and Methods

### Fly Stocks

For mutant analyses *sema 2a^03021^* [25], *sema 1a^P1^* [26], *plex A^Df(4)C3^* [47], and *plex B^KG00878^* [21],[48] were crossed into the *ppkEGFP* [9] stock. Stocks were made using GFP balancers [49]. Homozygous mutant embryos were identified by lack of GFP. For misexpression we used the following stocks: *UAS-robo3* [50],[51] inserts on second and third chromosome, *UAS-plexB* [21] and *UAS-plexA-HA* [47], *UAS-robo2* [51], *UAS-ephrin* [52],[53], *UAS-eph* [52], *UAS-unc5* [54], *UAS-frazzled* [55], *UAS-drl-DN* [56], *UAS-comm* [57], *UAS-robo*, 410 *EP*-lines from the Rorth collection [58],[59]. For the misexpression screen the UAS-lines were crossed into the *PO163GAL4, UAS-n-syb-GFP* stock [14],[60]. For rescue experiments the following embryos were analysed: *UAS-sema 1a*, *sema 1a^P1^*; *PO163GAL4*, *ppkEGFP* [26], *UAS-sema 1a, sema 1a^P1^*; *HB9GAL4, ppkEGFP*; *UAS-plexA-HA/+*; *PO163GAL4*, *ppkEGFP/+*; *plex A^Df(4)C3^*, and *UAS-plex B/PO163GAL4*, *ppkEGFP*; *plex B^KG00878^*. We also used *OK371GAL4* (gift of M. Landgraf), *GADGAL4* [29], *single mindedGAL4* [31], *UAS-reaper* [27] and *wnt5^D7^* stocks [56].

### Dissection

Embryos were staged and VNCs dissected out embryos as previously described [6],[61],[62]. For overexpression experiments embryos were grown at 29°C. VNCs were mounted with brain lobes down and VNC up to allow rapid, high-resolution, confocal imaging of transverse planes, perpendicular to the neuraxis.

### Immunocytochemistry

We used the following primary antibodies: anti-Sema 2a (MAb 19C2, developed by C. Goodman), anti-Slit (MAb C555.6D, developed by S. Artavanis-Tsakonas), anti-Fas II (MAb 1D4, developed by C. Goodman), and anti-Repo (MAb 8D12, developed by C. Goodman) supplied by the Developmental Studies Hybridoma bank (1∶10 dilution); anti-Sema 1a (1∶1,000 dilution, kindly provided by A. Kolodkin [26]; anti-Plex A (1∶500 dilution, kindly provided by L. Luo) [23], and Cy5-conjugated goat anti-horseradish peroxidase (1∶100 dilution; Jackson ImmunoResearch). Secondary antibodies were used at 1∶500 dilution: Alexa488-conjugated donkey anti-goat, Alexa488-conjugated goat anti-rabbit, Alexa633-conjugated goat anti-mouse, Alexa633-conjugated rabbit anti-mouse (Molecular Probes). Standard immunocytochemical procedures were followed [63], and immunofluorescence was visualised with Leica SP1 and Zeiss LSM confocal microscopes. Images are maximum projections of confocal z series processed with Adobe Photoshop software.

### Quantification Procedures

For quantification of Sema 2a gradients at 13-h AEL nine VNCs stained for Sema 2a were randomly chosen and A7 imaged using a Leica SP1. A confocal section was randomly chosen from each stack, the dorso-ventral axis manually drawn, and the neuropile was divided into nine equal dorso-ventral stripes, perpendicular to the midline and the average fluorescence intensity in each stripe was calculated. Values from different nerve cords were normalized such that the average intensity from each nerve cord was 1. For quantification of the Slit gradient at 13 h, 12 VNCs stained for Slit were randomly chosen, and A7 was imaged using a Leica SP1. A confocal section was randomly chosen from each stack, a line on either side of the midline was manually drawn, and the neuropile on either side of the midline was divided into four mediolateral stripes. The average fluorescence intensity in each stripe was calculated and normalised as above.

For a statistical analysis of defects in the pattern of sensory terminals (visualized with *PO163GAL4*, *UAS-n-syb-GFP*) along the medio-lateral axis we quantified the normalised surface area occupied by sensory terminals (sensory area, *SA*) in the medial domain of the neuropile (*SA*_M/T_ = *SA*[medial]/*SA*[medial+intermediate+lateral]) in randomly chosen transverse confocal sections from 30 different hemisegments for each genotype. Within a single embryo, we selected every tenth section (all confocal sections were 1-µm thick so that the analyzed sections were 10 µm apart from each other). A Student's *t*-test was used to compare the mean *SA*_M/T_ for the different genotypes.

For a statistical analysis of expansion or exclusion of sensory terminals (visualized with *PO163GAL4*, *UAS-n-syb-GFP*) into different dorso-ventral layers we compared *SA* in layer 2 (*SA*_2/h_ = *SA*(layer 2)/[hemisegment surface area]) or *SA* in layers 1, 3, and 4 (*SA*_1+3+4/h_ = *SA*[layer 1+3+4]/[hemisegment surface area]) in randomly chosen transverse confocal sections from more than 30 different hemisegments for each genotype. Within a single embryo, we selected every tenth section (all confocal sections were 1-µm thick so that the analyzed sections were 10 µm apart from each other). A Student's *t*-test was used to compare the mean *SA*_2/h_ or *SA*_1+3+4/h_ for the different genotypes.

For a statistical analysis of termination defects class IV md axons in the dorso-ventral axis, we quantified the percentage of hemisegments with aberrant terminals (Hat) in layers 1, 2, and 3 per embryo (per 14 hemisegments): Hat(1, 2, or 3) = (*n* hemisegments with terminals in 1, 2, or 3/14)×100 and the total percentage of hemisegments with aberrant terminals per embryo [Hat(1+2+3) = Hat(1)+Hat(2)+Hat(3)]. A Student's *t*-test was used to compare the mean Hat for the different genotypes. In some cases we also quantified the average (per embryo) relative proportion of hemisegments with aberrant terminal in each layer: Hat(1)/Hat(1+2+3), Hat(2)/Hat(1+2+3), and Hat(3)/Hat(1+2+3). We counted as “aberrant” only those hemisegments with terminals in layers 1, 2, or 3 (Figure S5A and S5B). We did not count as “aberrant” those axons that exhibit the *aberrant growth*, *with normal termination* phenotype (Figure S5C).

## Supporting Information

Figure S1 **Sensory neuron termination does not correlate with nerve route and position of entry into the neuropile.** (A) Diagram showing the pathways in the neuropile taken by sensory neurons that run in the ISN (magenta) and the SN (green) en route to their termination domains (yellow) in wild type (21 AEL), with respect to Fas II tracks (red). Diagram represents a projection of a confocal z series of transverse sections through an abdominal segment. Dorsal up. White arrowhead shows midline. Magenta lines indicate the pathways taken by ISN neurons in the neuropile. Green lines indicate the pathways taken by SN neurons in the neuropile. 2, 3, and 4 indicate sensory neuron termination domains in layers 2, 3, and 4, respectively. Scale bar: 10 mm. Sensory axons whose cell bodies are located ventrally in the body wall join the SN nerve, whereas axons whose cell bodies are located dorsally or laterally in the body wall join the ISN nerve. There is no correlation between the nerve that axons travel in and the position of their termination in the neuropile. Sensory axons running in the SN terminate in layers 2, 3, or 4, in correlation with their modality and dendritic morphology [9],[11]. For example, the ventral class IV neuron (vdaB) terminates in layer 4, the ventral ch neurons terminate in layer 3, and the ventral proprioceptive class I neuron vpda terminates in layer 2 (M. Zlatic, unpublished data) [9],[11]. Similarly, those sensory neurons that travel in the ISN terminate in layers 2, 3, or 4, depending on their modality and dendritic morphology. Dbd and the ddaE and ddaD class I md neurons, terminate in layer 2, the lateral ch neurons terminate in layer 3, and the dorsal class IV neuron ddaC terminates in layer 4 [9],[11]. Each of the three characteristic modality-specific sensory termination domains, therefore, contains some neurons that have travelled through the SN, and others that have travelled through the ISN. Thus, the position of termination in the neuropile does not seem to depend on the nerve through which the sensory neurons travel towards the neuropile nor on the position of entry into the neuropile. (B) Images show the patterns of growth and termination of md axons labelled with *109(280)GAL4, UAS-CD8GFPGFP* (white) in 21-h embryos. Dorsal is up. Red arrowheads show the midline. Magenta arrows indicate pathways taken by ISN neurons in the neuropile. Green arrows indicate pathways taken by SN neurons in the neuropile. 2 and 4 indicate md neuron termination domains in layers 2 and 4, respectively. Scale bar: 10 µm. Upper: a single section from a confocal z series through an abdominal segment showing the ISN pathways. Lower: a single section from the same series showing the SN pathways. Both ISN and SN md neurons terminate in layers 2 or 4, depending on their dendritic morphology.
(0.93 MB TIF)

Figure S2 **Distributions of Sema 2a and Sema 1a along the antero-posterior axis of the neuropile.** (A and B) Immunofluorescence visualisation of Sema 2a (A) and Sema 1a (B) (white) in *ppkeGFP* embryos (13-h AEL). Upper images show projections of confocal z series of longitudinal sections through the VNC. Central and lower images show single more dorsal and more ventral sections from the stack, respectively. Anterior is to the left. Red arrowheads show midline. a and p indicate the position of anterior (a) and posterior (p) commissures in each segment. Scale bar: 35 µm. (A) Levels of Sema 2a are uniform along the antero-posterior axis, thus Sema 2a is unlikely to provide instructive information for controlling neurite termination along this axis. Both in more dorsal (layer 2) and in more ventral (layer 3) longitudinal sections, levels of Sema 2a appear uniform along the antero-posterior axis. (B) Levels of Sema 1a are uniform along the antero-posterior axis. thus Sema 1a is unlikely to provide instructive information for controlling neurite termination along this axis. Both in more dorsal (layer 1) and in more ventral (layer 3) longitudinal sections, levels of Sema 1a appear uniform along the antero-posterior axis.
(1.34 MB TIF)

Figure S3 **Cellular sources of Sema 1a in the neuropile.** (A–E) Sema 1a is brought into dorsal neuropile, in part, by motor neurons. (A, C) Immunofluorescence visualisation of motor neuron dendrites labelled with *OK371GAL, UAS-CD8-GFP* control (white), in *OK371GAL4, UASCD8GFP* embryos (A) and in *OK371GAL4, UASCD8GFP, UAS-reaper* embryos (B) at 21-h AEL. (B, D) Immunofluorescence visualisation of Sema 1a pattern (white) in *OK371GAL4, UASCD8GFP* control (B) and *OK371GAL4, UAS-CD8GFP, UAS-reaper* (C) embryos 21-h AEL. Dorsal is up. Arrowheads indicate the midline. Magenta lines, neuropile boundaries. Red lines, layer boundaries. Numbers indicate layers. Scale bar: 10 µm. A. In control embryos processes of motor neurons labelled with *OK371GAL, UAS-CD8-GFP* are readily detectable and they are located in layer 1, which normally contains high levels of Sema1a. (B) In control embryos Sema 1a is present at high levels in layers 1 and 3. (C) Motor neuron dendrites are not detectable in *OK371GAL4, UAS-CD8GFP, UAS-reaper.* (D) Sema 1a expression in the same animal, as in (C). Sema 1a levels in layer 1 are reduced relative to layer 3 in the absence of motor neuron dendrites. (E) Quantification of Sema 1a levels in layer 1 relative to layer 3 in the same hemisegment, for *OK371-GAL4, UASCD8GFP* control and *OK371GAL4, UAS-CD8GFP, UAS-reaper*, 21-h old embryos. For this purpose, embryos of the two genotypes were stained with antibody against GFP (to distinguish between embryos with and without motor neurons). In each embryo seven sections from seven different hemisegments where chosen at random, and for each section the ratio of the pixel intensity (PI) for the channel showing Sema 1a staining in layer 1 relative to layer 3 (*PI*_1/3_ = *PI*[Sema 1a in layer 1]/*PI*[Sema 1a in layer 3]) was calculated. A significant decrease (*p* = 2×10^−6^; Student's *t*-test) in pixel intensity in layer 1 relative to layer 3 was observed in *OK371GAL4, UAS-CD8GFP, UAS-reaper* embryos (average *PI*_1/3_ = 0.73; standard deviation [SD] = 0.09, *n* = 65 hemisegments) compared to *OK371GAL4, UAS-CD8GFP* controls (average *PI*_1/3_ = 0.81; SD = 0.08, *n* = 48 hemisegments). (F and G) Sema 1a is brought into dorsal and ventral neuropile, in part, by GABAergic interneurons. (F and G) Immunofluorescence visualisation of motor neurons and GABAergic processes labelled with *GADGAL, UAS-CD8-GFP* (white) (F) and of Sema 1a pattern (white) in *GADGAL4, UAS-CD8GFP, UAS-reaper* (G), 21-h old embryos. Dorsal is up. Arrowheads indicate the midline. Magenta lines, neuropile boundaries. Red lines, layer boundaries. Numbers indicate layers. M, medial; I, intermediate; L, lateral domains. Scale bar: 10 µm. (F) Processes of GABAergic interneurons and motor neurons together (white) cover the dorsal and central regions of the neuropile, which normally contain high levels of Sema 1a. (G) Sema 1a (white) levels appear highly reduced in embryos that lack both GABAergic interneurons and motor neurons. The characteristic Sema 1a distribution pattern is no longer detectable.
(1.73 MB TIF)

Figure S4 **Restoration of** ***sema 2a*** **in midline cells partially rescues the aberrant central projection of Class IV axons.** (A–C) Immunofluorescence visualisation of Sema 2a (white), in *sema 2a* mutant (A) and in *sema 2a, UAS-sema 2a; single-mindedGAL4, ppkeGFP* (B and C) embryos (21-h old). (D) Projections of class IV axons (green) and Fas II tracts (red) in *sema 2a,UAS-sema 2a;single-mindedGAL4,ppkeGFP* embryos (21-h old). (A and B) Image shows projections of a confocal z series of longitudinal sections through the VNC. Anterior is to the left. Red arrowheads show midline. Magenta line: neuropile boundary. Scale bar: 14 µm. (C and D) Images show projections of a confocal z series of transverse sections through A7. Dorsal is up. Arrowheads show midline. Magenta line (C): neuropile boundary. Red (C) and white (D) lines, layer boundaries. Numbers indicate layers: M, medial; I, intermediate; L, lateral domains. Scale bar: 9 µm. (A) Sema 2a expression is not detectable above background levels in the neuropile of 21-h-old *sema 2a* mutant embryos. (B) High levels of Sema 2a expression are detectable in *sema 2a, UAS-sema 2a; single-mindedGAL4, ppkeGFP embryos.* (C) Transverse view of Sema 2a expression in *sema 2a, UAS-sema 2a; single-mindedGAL4, ppkeGFP embryos.* Sema 2a expression in midline cells, in an otherwise mutant background, results in its distribution throughout the neuropile, with lower levels detectable on the lateral edges of the neuropile and in the ventral-most neuropile. (D) Restoration of Sema 2a expression in midline cells alone reduces the aberrant central projection of class IV neurons (compare to Figure 5C). However, this rescue was accompanied by additional defects in the medio-lateral axis, with increased lateral termination of class IV axons. Thus the source of the Sema 2a gradients could potentially be a subset of the midline cells, although we cannot exclude the possibility that some other cells are the endogenous source of this cue in the CNS. (E) Chart shows average percentage of hemisegments per embryo (per 14 abdominal hemisegments) with aberrant class IV terminals in layer 2 (Hat(2)), in *sema 2a*/+, *sema 2a* mutant and *sema 2a*,*UAS-sema 2a;single-mindedGAL4*,*ppkeGFP* embryos. Hat(2) is significantly higher in *sema 2a* (*p* = 4×10^−4^; Student's *t*-test; average Hat(2) = 3.3%; *n* = 24 embryos, 336 hemisegments), than in *sema 2a/+* controls (average Hat(2) = 0%, *n* = 30 embryos, 420 hemisegments). *sema 2a*, *UAS-sema 2a*; *sim-GAL4*,*ppkeGFP* show a significant reduction in Hat(2) (red **, *p* = 0.006; Student's *t*-test; average Hat(2) = 0.7%, *n* = 29 embryos, 406 hemisegments), compared to *sema 2a* mutant embryos (average Hat(2) = 3.3%).
(1.28 MB TIF)

Figure S5 **Examples of growth and termination errors of class IV axons in mutant embryos.** Projections of class IV axons (white) in mutant embryos (21-h AEL). Images show projections of a confocal z series of transverse sections through A7. Dorsal is up. Arrowheads show midline. Magenta arrows point to aberrant (dorsal or central) terminals of class IV axons. Green arrows point to class IV axons that initially grow normally (ventrally) in the neuropile, but afterwards turn dorsally, and terminate in aberrant layers (1, 2, or 3). Red arrows point to class IV axons that grow aberrantly in dorsal or central neuropile. We define terminals as large structures that form at the tips of axons (although sometimes they form along the axon path, on either side of the main axon trunk, which continues growing). These structures are thicker than the axon itself and we assume they contain presynaptic specialisations. Class IV axons exhibit several kinds of phenotypes in *sema* and *plex* mutant embryos. The most striking is *normal initial growth with aberrant termination*, where the axon initially grows appropriately towards its target area in the ventral medial neuropile, but then makes a sharp dorsal turn, and terminates in layers 1, 2, or 3 (for examples see Figures 5D, left axons, 7E, right axon, and S5A). In the case of *aberrant growth, with aberrant termination*, the misrouted axon grows through and forms terminals in layers 1, 2, or 3 (for examples see Figures 5B, 6F, 7B, 7D, right axon, and S5B). Some of these axons never reach their wild-type layer 4 (Figures 5B, 6F, 7B, and S5B) while others send a branch ventrally, after they have formed a terminal dorsally or centrally (right axon in Figure 7D). In the case of *aberrant growth, with normal termination*, the misrouted axon turns ventrally after growing through dorsal or central layers and terminates in its wild-type layer 4 (for example see Figures 7D, left axon, and S5C). For all our statistical analysis of termination defects (see below) we counted as “aberrant” only those hemisegments that exhibit the *aberrant termination* phenotypes (Figure S5A and S5B), but we counted as “normal” those hemisegments that exhibit the *aberrant growth, with normal termination* phenotype (Figure S5C). (A) Examples of *normal initial growth with aberrant termination,* where class IV axons grew in normally, and afterwards aberrantly turned dorsally or centrally and terminated there, in *sema1a, sema 2a* double mutant embryos (21 AEL). These examples show that the position of entry does not determine the position of termination. Despite the fact that these axons initially grow appropriately in the ventral neuropile, they afterwards alter direction of growth and invade aberrant neuropile layers, where they terminate. (B) Example of class IV axons showing both *aberrant growth and aberrant termination* in a *sema 1a* mutant embryo. The axons initially grow in dorsal neuropile, where they also forms a terminal at the midline. (C) Example of class IV axons showing *aberrant growth, but normal termination* in a *plex B* mutant embryo. The axon grows through dorsal neuropile, but turns ventrally at the midline, without forming a terminal. It forms a terminal once it reaches the ventral neuropile, in its appropriate location.
(1.35 MB TIF)

Figure S6 **Defective positioning of Fas II tracts in different mutant backgrounds.** Graphs show percentages of segments (*n* = 175) in which L1 (blue), I2 (green), I3 (yellow), M1 (black), and M2 (white) tracts project aberrantly in *sema 1a^P1^/+* (A), *sema 1a^P1^* (B), *sema 2a* (C), and *sema 1a^P1^*, *sema 2a* double mutant (D) 21-h embryos. (A) In *sema 1a^P1^/+* control embryos Fas II tracts grow normally in the dorso-ventral axis. In both *sema 1a^P1^* and *sema 2a* embryos Fas II tracts are affected (B and C) and the disruption is more severe in double mutants (D).
(0.29 MB TIF)

Figure S7 **Role of Semas in patterning the antero-posterior axis of the neuropile.** We assessed the potential role of Sema 1a and Sema 2a in controlling termination of class IV axons in the antero-posterior axis by analysing their projections in a top-down view of the neuropile in wild type and in *sema 1a, sema 2a* double mutants (see Figure S5A and S5B). We chose the *sema 1a, sema 2a* double mutant for this analysis, because it exhibited the strongest phenotypes in the dorso-ventral axis. (A and B) Projections of class IV axons labelled with *ppkEGFP* (white) in *sema 1a/+; ppkEGFP* control (A) and *sema 1a, sema 2a; ppkEGFP* (B), 21-h embryos. Images show projections of confocal z series of longitudinal sections of the VNC (from T1 to A4). Anterior left. Arrowheads show midline. a, anterior half of the segment; p, posterior half of the segment. Scale bar: 16 µm. (A) Top-down view of wild-type class IV projections in T1–A4. Wild-type class IV axons grow asymmetrically, within their normal ventral and medial termination domain, forming a thick anterior branch and very thin processes that extend posteriorly. (B) Top-down view of class IV projections in T1–A4 in *sema 2a, sema 1a* double mutants. Note that while the class IV terminals appear disorganised compared to wild type, they still appear asymmetric and largely confined to the anterior portion of the segment. We assume the observed disorganization is a consequence of the major defects in growth and termination in the dorsoventral axis. (C) Quantification of the average surface area occupied by class IV terminals in the posterior half of the hemisegment, relative to the total surface area covered by class IV terminals in a hemisegment [*SA*_p/(p+a)_ = *SA*(posterior)/*SA*(posterior+anterior]. Quantification of *SA*_p/(p+a)_ does not reveal a significant increase (*p* = 0.09; Student's *t*-test average *SA*_p/(p+a)_ = 0.22; SD = 0.16; *n* = 50 hemisegments) for the double mutants with respect to wild-type embryos (average *SA*_p/(p+a)_ = 0.19; SD = 0.1; *n* = 55 hemisegments). For comparison we analyzed the antero-posterior distribution of class IV projections in embryos mutant for the gene *wnt 5*, that has previously been implicated in controlling axon projections in the antero-posterior axis [56]. Wnt 5 is secreted by neurites in the posterior commissure of each segment. We also analyzed class IV projections in embryos in which the dominant negative form of Derailed (Drl) has been selectively targeted to sensory neurons *(PO163GAL4,UAS-DN-drl)*. The Wnt 5 receptor Drl is present on the growth cones and axons of neurons crossing in the anterior commissure and is required to prevent these cells from crossing aberrantly in the posterior commissure [56]. In contrast, to the *sema1a, sema 2a* double mutants, when we analysed class IV projections in *wnt 5^D7^* mutants and in *PO163GAL4,UAS-DN-drl* embryos, we did find a significant increase in *SA*_p/(p+a)_ compared to wild type (for *wnt 5*, *p* = 1.8×10^−7^; Student's *t*-test; average *SA*_p/(p+a)_ = 0.34; SD = 0.12; *n* = 16 hemisegments; for *PO163-DN-drl*, *p* = 2.2×10^−7^; Student's *t*-test; average *SA*_p/(p+a)_ = 0.33; SD = 0.12; *n* = 30 hemisegments). Thus Sema 1a and Sema 2a do not appear to play a major role in confining class IV terminals to the anterior portion of the segment.
(1.15 MB TIF)

Figure S8 **Plexin expression in sensory neurons (A, B, and D) and in the CNS (C).** (A and B) Immunofluorescence visualisation of sensory neuron cell bodies labelled with antibody against horseradish peroxidase (HRP) (A) or PPK-EGFP (red) (in B) and Plex A (A and B) at 13-h AEL. Dorsal is up. (A) Plex A expression (white in ii and green in iii) in dorsal (d) and lateral (l) clusters of sensory neurons (white in i and red in iii). Strong Plex A expression is visible in sensory neuron cell bodies in both clusters. Scale bar: 15 µm. (B) Plex A protein (white in ii and green in iii) is strongly expressed in class IV md neuron cell bodies (white in i and red in iii). Scale bar: 10 µm. (C) Immunofluorescence visualisation of Plex A protein (white in ii and green in iii) in a transverse section of the neuropile labelled with HRP (white in i and red in iii) at 13-h AEL. Image shows a projection of a confocal z series of 1-mm thick transverse sections through abdominal segment A7. Dorsal is up. Arrowheads show the midline. Outlines indicate neuropile boundaries. Scale bar: 5 mm. (D) In situ hybridisation showing *plex B* mRNA expression in dorsal (d) and lateral (l) clusters of sensory neurons in the embryonic body wall. Dorsal is up. Scale bar: 20 µm. In situ hybridization protocol: DIG-labelled RNA antisense and sense probes were generated with the Ambion Megascript kit and DIG-UTP (purchased from Roche), following the manufacturer's instructions. In situ hybridization was performed according to a protocol kindly provided by Nipam Patel (University of California, Berkeley). DNA templates for in vitro transcription: DNA fragments were amplified by PCR with Primer1 (GCGCGCGTAATACGACTCACTATAGGG) and Primer2 (GCGCGCAATTAACCCTCACTAAAGGG) from pBluescript(SK)-PlexinB-CK00213 (AA142091) (EST, ∼1.7-kb insert) using the following key PCR parameters: annealing at 66°C, 5 min extension at 72°C, 30 cycles; Primer1 and Primer2 include the T7 and T3 promoter sequences. In vitro transcription: plex B: T3 (antisense), T7 (sense).
(3.90 MB TIF)

Figure S9 **Plex B and Plex A prevent expansion of sensory terminals into regions with high Sema 1a levels. (**A–D) Representative images of sensory terminals labelled with *PO163GAL4*, *UAS-n-syb-GFP* (white) in 21-h embryos (left) and diagrams showing patterns of sensory terminals superimposed for different genotypes (right). In all cases images show projections of a confocal z series of transverse sections through A7. Dorsal is up. Arrowheads show midline. White lines, layer boundaries. Numbers indicate layers: M, medial; I, intermediate; L, lateral domains. Scale bar: 10 µm. (A) Expressing Plex B in sensory neurons in a wild-type background results in exclusion of sensory neuron terminals from neuropile layer 2 (see also Figure 2C). Quantification of *SA*_2/h_ (*SA*_2/h_ = *SA*(layer 2)/[hemisegment surface area]) reveals a significant decrease (***, *p* = 4×10^−17^; Student's *t*-test; average *SA*_2/h_ = 0.003; SD = 0.006; *n* = 30 hemisegments) with respect to wild-type embryos (average *SA*_2/h_ = 0.06; SD = 0.02; *n* = 30 hemisegments). However, in these embryos, ectopic sensory terminals in layer 1 still remain largely excluded from intermediate and lateral portions of layer 1, which contain highest Sema 1a levels. Right: Diagram showing the pattern of Plex B expressing sensory terminals (green) superimposed on the wild-type pattern (yellow). (B) Expressing Plex B in sensory neurons in a *sema 1a* mutant background still excludes sensory terminals from neuropile layer 2. However, in these embryos ectopic sensory terminals invade the entire layer 1 and are no longer excluded from its lateral portions, which normally contain highest Sema 1a levels. This results in an overall increase in the surface area occupied by sensory neuron terminals in layers 1 and 3. Right: Diagram showing the patterns of Plex B expressing sensory terminals in *sema 1a* mutant (green) and wild-type (yellow) backgrounds, superimposed. Quantification reveals a significant increase in *SA*_1+3+4/h_ (*SA*_1+3+4/h_ = *SA*[layer 1+3+4]/[hemisegment surface area]) (***, *p* = 4×10^−5^; Student's *t*-test) when Plex B is overexpressed in sensory neurons in a *sema 1a* mutant background (average *SA*_1+3+4/h_ = 0.6 and SD = 0.08, *n* = 22 hemisegments), compared to embryos in which Plex B is overexpressed in wild-type background (average *SA*_1+3+4/h_ = 0.5; SD = 0.06; *n* = 32 hemisegments). (C) In *plex A* mutant embryos, sensory terminals aberrantly invade neuropile layers 1, 3, and to a lesser extent layer 2. As in the case of *sema 1a* mutant embryos, this results in an overall increase in the surface area occupied by sensory neuron terminals in layers 1 and 3. Right: Diagram showing the patterns of sensory terminals in *plex A* mutant (green) and wild-type (yellow) backgrounds, superimposed. Quantification reveals a significant increase of *SA*_1+3+4/h_ (***, *p* = 6×10^−34^; Student's *t*-test) in *plex A* mutants (average *SA*_1+3+4/h_ = 0.7 and SD = 0.08, *n* = 62 hemisegments), with respect to wild type (average *SA*_1+3+4/h_ = 0.3; SD = 0.05; *n* = 32 hemisegments). (D) Expressing Plex B in sensory neurons in a *plex A* mutant background is sufficient to exclude sensory terminals from lateral and intermediate portions of neuropile layer 1. In these embryos ectopic sensory terminals do not invade regions of layer 1, which contain highest Sema 1a levels. As a result, sensory neuron terminals in layers 1 and 3 occupy a smaller surface area than in *plex A* mutants. Thus, in the absence of Plex A, Plex B is sufficient to exclude sensory terminals from regions of highest Sema 1a expression. Plex B may therefore function as a receptor for Sema 1a. Right: Diagram showing superimposed patterns of sensory terminals with and without Plex B expression in *plex A* mutants. Terminals with Plex B expression: green. Terminals without Plex B expression: yellow. Quantification reveals a significant decrease of *SA*_1+3+4/h_ (***, *p* = 4×10^−9^; Student's *t*-test) when Plex B is expressed in sensory terminals in a *plex A* mutant background (*SA*_1+3+4/h_ = 0.5 and SD = 0.1, *n* = 55 hemisegments), compared to *plex A* mutants (*SA*_1+3+4/h_ = 0.7 and SD = 0.08, *n* = 62 hemisegments). (E and F) Bar charts show the average *SA*_1+3+4/h_ under different conditions as indicated. (E) The average *SA*_1+3+4/h_ (average *SA*_1+3+4/h_ = 0.6, SD = 0.08, *n* = 22 hemisegments) is significantly higher (***, *p* = 4×10^−5^; Student's *t*-test), when Plex B is expressed in a *sema 1a* mutant background, compared to its expression in wild-type background (average *SA*_1+3+4/h_ = 0.5; SD = 0.06; *n* = 32 hemisegments). (F) The average *SA*_1+3+4/h_ (average *SA*_1+3/h_ = 0.5 and SD = 0.1, *n* = 55 hemisegments) is significantly lower (***, *p* = 4×10^−9^; Student's *t*-test) for sensory terminals that express Plex B in a *plex A* mutant background, compared to *plex A* mutants (average *SA*_1+3+4/h_ = 0.7 and SD = 0.08, *n* = 62 hemisegments). In contrast, we did not observe a significant difference (*p* = 0.6; Student's *t*-test) between the *SA*_1+3+4/h_ of sensory terminals that express Plex B in a *plex A* mutant background (average *SA*_1+3+4/h_ = 0.5 and SD = 0.1, *n* = 55 hemisegments) and those that express Plex B in wild-type background (*SA*_1+3+4/h_ = 0.5; SD = 0.06; *n* = 32 hemisegments).
(1.24 MB TIF)

Figure S10 **Fas II defects are not rescued by selective restoration of Plex B expression in sensory neurons.** Graphs show percentage of segments (*n* = 175) in which L1 (blue), I2 (green), I3 (yellow), M1 (black), M2 (white) tracts project aberrantly. (A) In *ppkEGFP; plexB* embryos Fas II tracts are severely affected. (B) When Plex B expression is selectively restored in sensory neurons alone, in *UAS-plexB;PO163GAL4,ppkEGFP;plexB* embryos, Fas II tracts continue to exhibit the mutant phenotype.
(0.19 MB TIF)

Table S1 **Results of the misexpression screen.** We identified 11 genes (2.6%) that change the pattern of sensory terminals, without producing pronounced changes in neuron number or preventing sensory axons from reaching the CNS. In these experiments, sensory terminals shift independently of Fas II tracts, which remain in their wild-type position and relation to each other. Of the 11 genes, two produced specific shifts along the dorso-ventral axis. The table gives the list of 11 genes, which, when misexpressed in sensory neurons alone, produce specific shifts in the dorso-ventral, medio-lateral or antero-posterior axes.
(0.04 MB DOC)

## Abbreviations

AEL: after egg laying
ch: chordotonal
CNS: central nervous system
dbd: dorsal bipolar dendritic
Fas II: Fasciclin II
Hat: number of hemisegments with aberrant termination of class IV md axons
ISN: intersegmental nerve
md: multidendritic
Plex A: Plexin A
Plex B: Plexin B
Sema 1a: Semaphorin 1a
Sema 2a: Semaphorin 2a
SA: sensory area (surface area occupied by sensory terminals in cross section)
SD: standard deviation
SN: segmental nerve
VNC: ventral nerve cord
