# Supplementary figures and images for: Positional Cues in the Drosophila Nerve Cord: Semaphorins Pattern the Dorso-Ventral Axis
Source: PLoS Biol. 2009 Jun 23;7(6):e1000135. doi: 10.1371/journal.pbio.1000135 (PMC2690435; doi:10.1371/journal.pbio.1000135)

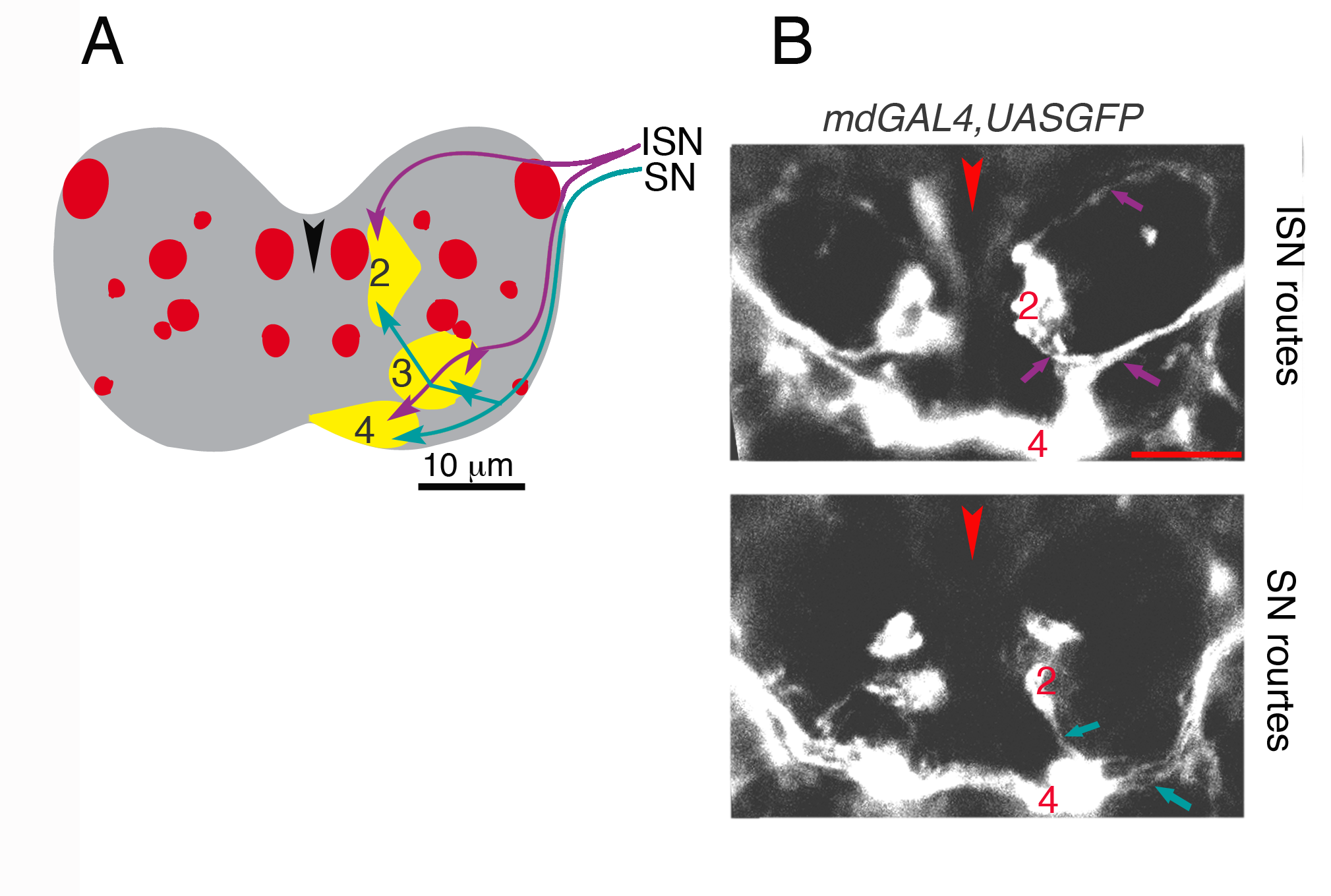

Supplement: Figure S1 — Sensory neuron termination does not correlate with nerve route and position of entry into the neuropile. (A) Diagram showing the pathways in the neuropile taken by sensory neurons that run in the ISN (magenta) and the SN (green) en route to their termination domains (yellow) in wild type (21 AEL), with respect to Fas II tracks (red). Diagram represents a projection of a confocal z series of transverse sections through an abdominal segment. Dorsal up. White arrowhead shows midline. Magenta lines indicate the pathways taken by ISN neurons in the neuropile. Green lines indicate the pathways taken by SN neurons in the neuropile. 2, 3, and 4 indicate sensory neuron termination domains in layers 2, 3, and 4, respectively. Scale bar: 10 mm. Sensory axons whose cell bodies are located ventrally in the body wall join the SN nerve, whereas axons whose cell bodies are located dorsally or laterally in the body wall join the ISN nerve. There is no correlation between the nerve that axons travel in and the position of their termination in the neuropile. Sensory axons running in the SN terminate in layers 2, 3, or 4, in correlation with their modality and dendritic morphology [9],[11]. For example, the ventral class IV neuron (vdaB) terminates in layer 4, the ventral ch neurons terminate in layer 3, and the ventral proprioceptive class I neuron vpda terminates in layer 2 (M. Zlatic, unpublished data) [9],[11]. Similarly, those sensory neurons that travel in the ISN terminate in layers 2, 3, or 4, depending on their modality and dendritic morphology. Dbd and the ddaE and ddaD class I md neurons, terminate in layer 2, the lateral ch neurons terminate in layer 3, and the dorsal class IV neuron ddaC terminates in layer 4 [9],[11]. Each of the three characteristic modality-specific sensory termination domains, therefore, contains some neurons that have travelled through the SN, and others that have travelled through the ISN. Thus, the position of termination in the neuropile does not s [file pbio.1000135.s001.tif]

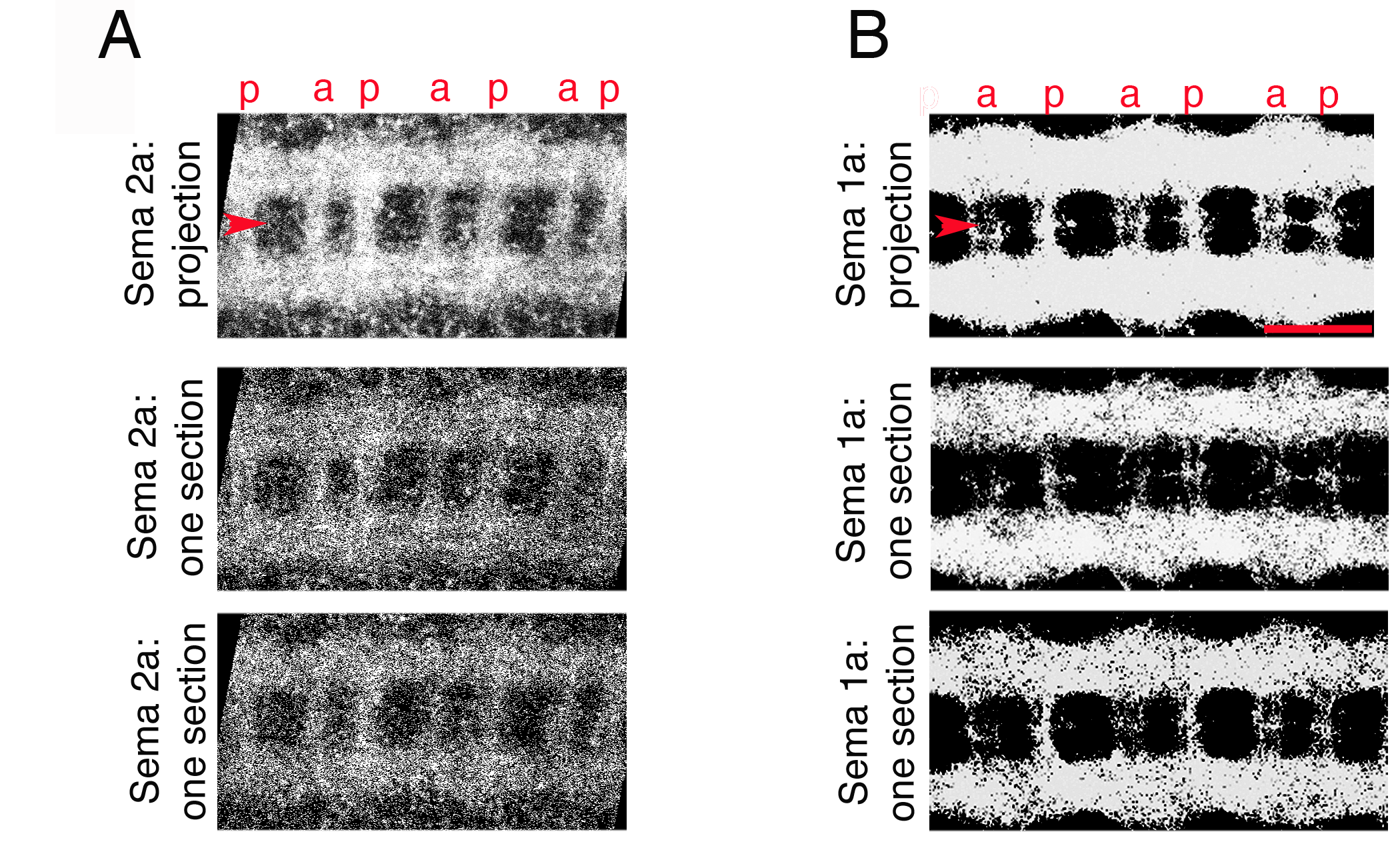

Supplement: Figure S2 — Distributions of Sema 2a and Sema 1a along the antero-posterior axis of the neuropile. (A and B) Immunofluorescence visualisation of Sema 2a (A) and Sema 1a (B) (white) in ppkeGFP embryos (13-h AEL). Upper images show projections of confocal z series of longitudinal sections through the VNC. Central and lower images show single more dorsal and more ventral sections from the stack, respectively. Anterior is to the left. Red arrowheads show midline. a and p indicate the position of anterior (a) and posterior (p) commissures in each segment. Scale bar: 35 µm. (A) Levels of Sema 2a are uniform along the antero-posterior axis, thus Sema 2a is unlikely to provide instructive information for controlling neurite termination along this axis. Both in more dorsal (layer 2) and in more ventral (layer 3) longitudinal sections, levels of Sema 2a appear uniform along the antero-posterior axis. (B) Levels of Sema 1a are uniform along the antero-posterior axis. thus Sema 1a is unlikely to provide instructive information for controlling neurite termination along this axis. Both in more dorsal (layer 1) and in more ventral (layer 3) longitudinal sections, levels of Sema 1a appear uniform along the antero-posterior axis. (1.34 MB TIF) [file pbio.1000135.s002.tif]

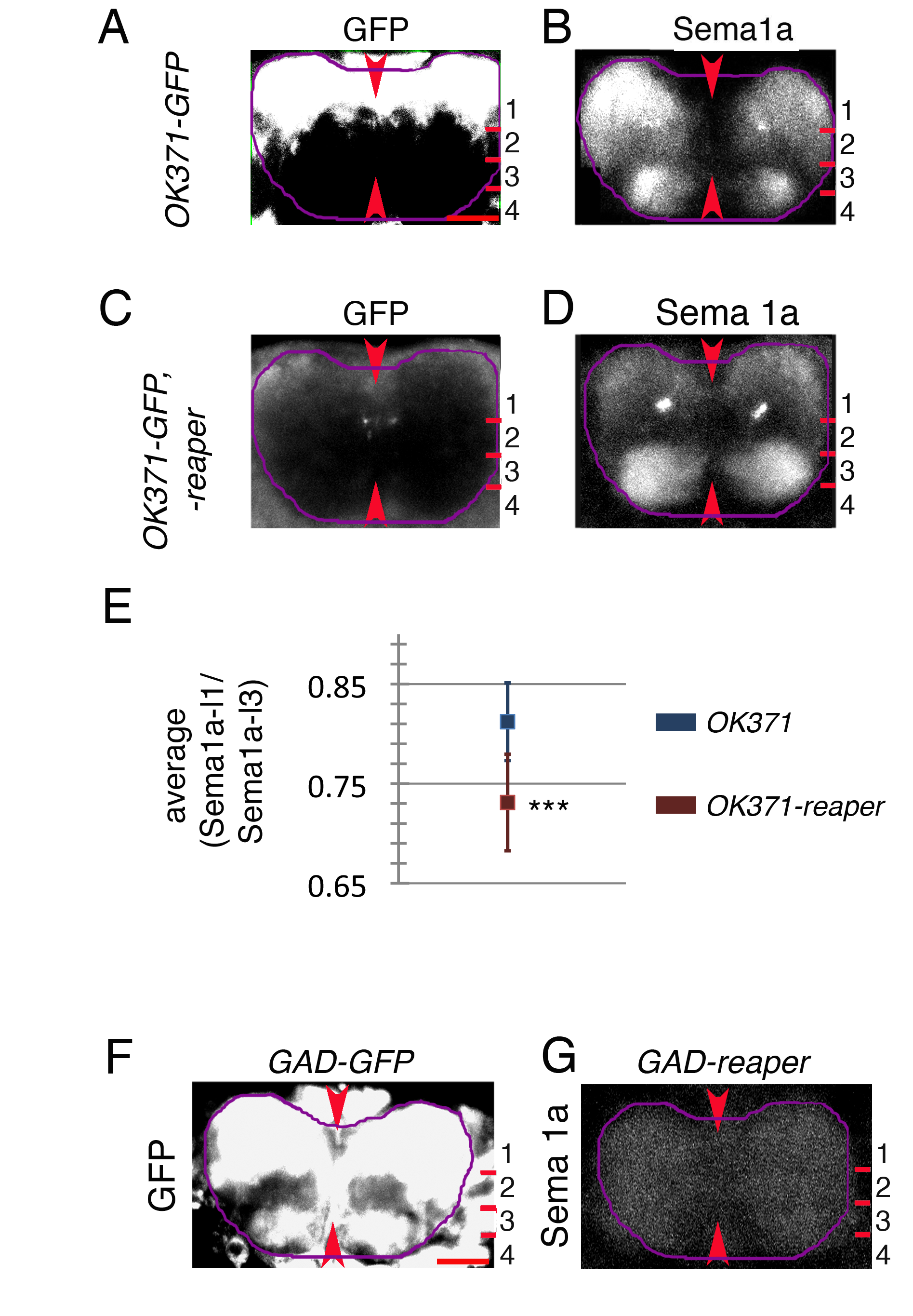

Supplement: Figure S3 — Cellular sources of Sema 1a in the neuropile. (A–E) Sema 1a is brought into dorsal neuropile, in part, by motor neurons. (A, C) Immunofluorescence visualisation of motor neuron dendrites labelled with OK371GAL, UAS-CD8-GFP control (white), in OK371GAL4, UASCD8GFP embryos (A) and in OK371GAL4, UASCD8GFP, UAS-reaper embryos (B) at 21-h AEL. (B, D) Immunofluorescence visualisation of Sema 1a pattern (white) in OK371GAL4, UASCD8GFP control (B) and OK371GAL4, UAS-CD8GFP, UAS-reaper (C) embryos 21-h AEL. Dorsal is up. Arrowheads indicate the midline. Magenta lines, neuropile boundaries. Red lines, layer boundaries. Numbers indicate layers. Scale bar: 10 µm. A. In control embryos processes of motor neurons labelled with OK371GAL, UAS-CD8-GFP are readily detectable and they are located in layer 1, which normally contains high levels of Sema1a. (B) In control embryos Sema 1a is present at high levels in layers 1 and 3. (C) Motor neuron dendrites are not detectable in OK371GAL4, UAS-CD8GFP, UAS-reaper. (D) Sema 1a expression in the same animal, as in (C). Sema 1a levels in layer 1 are reduced relative to layer 3 in the absence of motor neuron dendrites. (E) Quantification of Sema 1a levels in layer 1 relative to layer 3 in the same hemisegment, for OK371-GAL4, UASCD8GFP control and OK371GAL4, UAS-CD8GFP, UAS-reaper, 21-h old embryos. For this purpose, embryos of the two genotypes were stained with antibody against GFP (to distinguish between embryos with and without motor neurons). In each embryo seven sections from seven different hemisegments where chosen at random, and for each section the ratio of the pixel intensity (PI) for the channel showing Sema 1a staining in layer 1 relative to layer 3 (PI 1/3 = PI[Sema 1a in layer 1]/PI[Sema 1a in layer 3]) was calculated. A significant decrease (p = 2×10^−6^; Student's t-test) in pixel intensity in layer 1 relative to layer 3 was observed in OK371GAL4, UAS-CD8GFP, UAS-reaper embryos (average PI 1/3 = 0.73; standard deviation [SD] = [file pbio.1000135.s003.tif]

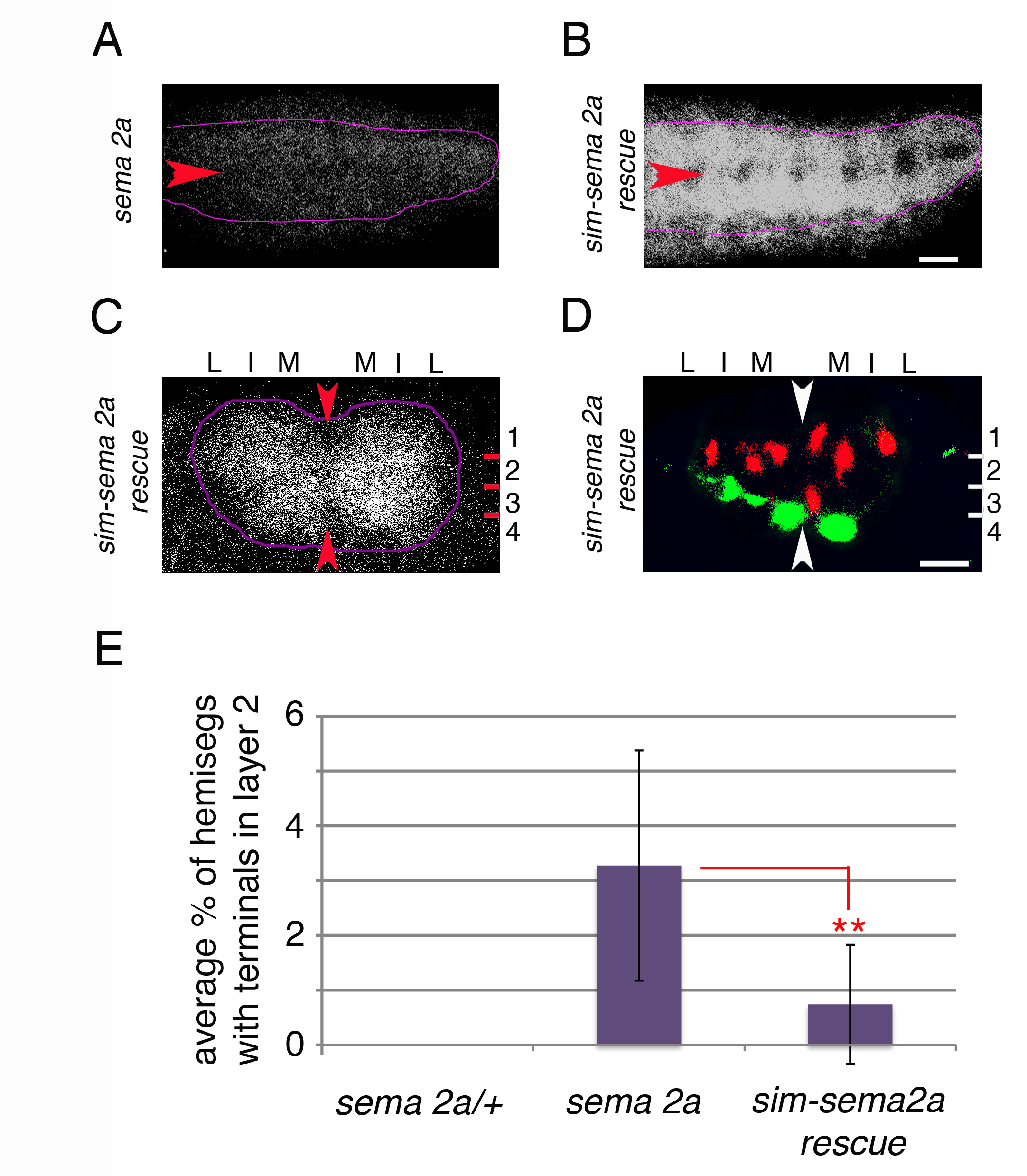

Supplement: Figure S4 — Restoration of sema 2a in midline cells partially rescues the aberrant central projection of Class IV axons. (A–C) Immunofluorescence visualisation of Sema 2a (white), in sema 2a mutant (A) and in sema 2a, UAS-sema 2a; single-mindedGAL4, ppkeGFP (B and C) embryos (21-h old). (D) Projections of class IV axons (green) and Fas II tracts (red) in sema 2a,UAS-sema 2a;single-mindedGAL4,ppkeGFP embryos (21-h old). (A and B) Image shows projections of a confocal z series of longitudinal sections through the VNC. Anterior is to the left. Red arrowheads show midline. Magenta line: neuropile boundary. Scale bar: 14 µm. (C and D) Images show projections of a confocal z series of transverse sections through A7. Dorsal is up. Arrowheads show midline. Magenta line (C): neuropile boundary. Red (C) and white (D) lines, layer boundaries. Numbers indicate layers: M, medial; I, intermediate; L, lateral domains. Scale bar: 9 µm. (A) Sema 2a expression is not detectable above background levels in the neuropile of 21-h-old sema 2a mutant embryos. (B) High levels of Sema 2a expression are detectable in sema 2a, UAS-sema 2a; single-mindedGAL4, ppkeGFP embryos. (C) Transverse view of Sema 2a expression in sema 2a, UAS-sema 2a; single-mindedGAL4, ppkeGFP embryos. Sema 2a expression in midline cells, in an otherwise mutant background, results in its distribution throughout the neuropile, with lower levels detectable on the lateral edges of the neuropile and in the ventral-most neuropile. (D) Restoration of Sema 2a expression in midline cells alone reduces the aberrant central projection of class IV neurons (compare to Figure 5C). However, this rescue was accompanied by additional defects in the medio-lateral axis, with increased lateral termination of class IV axons. Thus the source of the Sema 2a gradients could potentially be a subset of the midline cells, although we cannot exclude the possibility that some other cells are the endogenous source of this cue in the CNS. (E) Chart shows averag [file pbio.1000135.s004.tif]

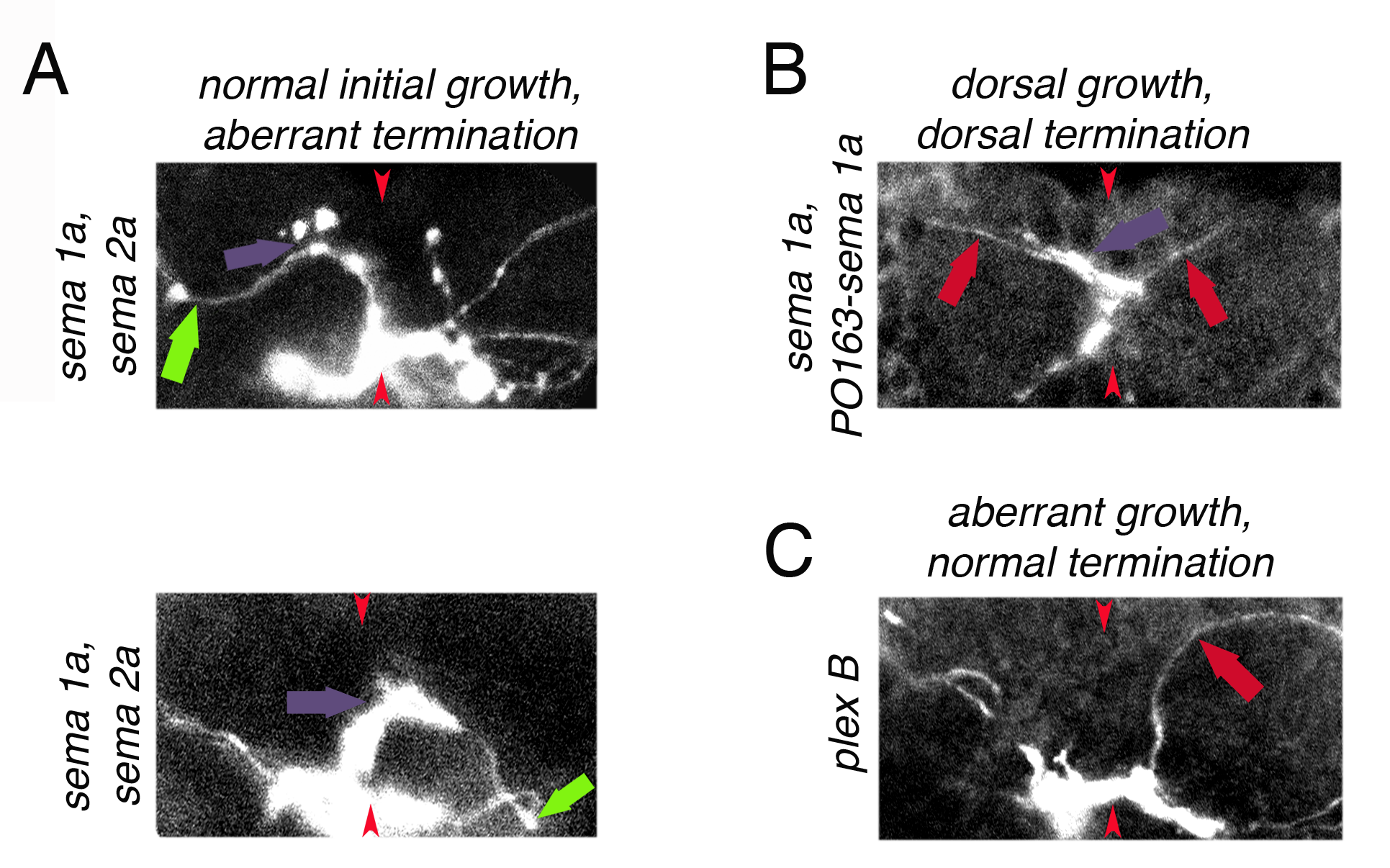

Supplement: Figure S5 — Examples of growth and termination errors of class IV axons in mutant embryos. Projections of class IV axons (white) in mutant embryos (21-h AEL). Images show projections of a confocal z series of transverse sections through A7. Dorsal is up. Arrowheads show midline. Magenta arrows point to aberrant (dorsal or central) terminals of class IV axons. Green arrows point to class IV axons that initially grow normally (ventrally) in the neuropile, but afterwards turn dorsally, and terminate in aberrant layers (1, 2, or 3). Red arrows point to class IV axons that grow aberrantly in dorsal or central neuropile. We define terminals as large structures that form at the tips of axons (although sometimes they form along the axon path, on either side of the main axon trunk, which continues growing). These structures are thicker than the axon itself and we assume they contain presynaptic specialisations. Class IV axons exhibit several kinds of phenotypes in sema and plex mutant embryos. The most striking is normal initial growth with aberrant termination, where the axon initially grows appropriately towards its target area in the ventral medial neuropile, but then makes a sharp dorsal turn, and terminates in layers 1, 2, or 3 (for examples see Figures 5D, left axons, 7E, right axon, and S5A). In the case of aberrant growth, with aberrant termination, the misrouted axon grows through and forms terminals in layers 1, 2, or 3 (for examples see Figures 5B, 6F, 7B, 7D, right axon, and S5B). Some of these axons never reach their wild-type layer 4 (Figures 5B, 6F, 7B, and S5B) while others send a branch ventrally, after they have formed a terminal dorsally or centrally (right axon in Figure 7D). In the case of aberrant growth, with normal termination, the misrouted axon turns ventrally after growing through dorsal or central layers and terminates in its wild-type layer 4 (for example see Figures 7D, left axon, and S5C). For all our statistical analysis of termination defects (see below) [file pbio.1000135.s005.tif]

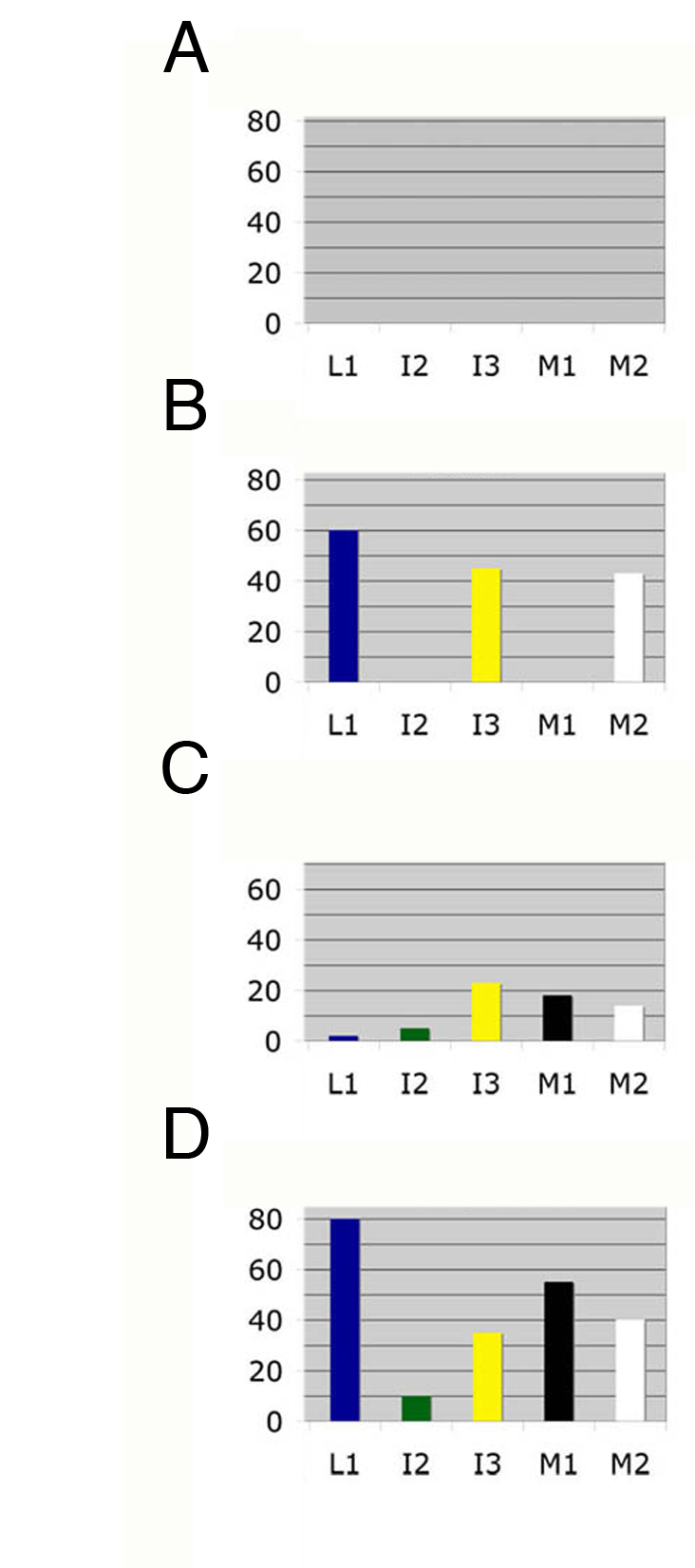

Supplement: Figure S6 — Defective positioning of Fas II tracts in different mutant backgrounds. Graphs show percentages of segments (n = 175) in which L1 (blue), I2 (green), I3 (yellow), M1 (black), and M2 (white) tracts project aberrantly in sema 1aP1/+ (A), sema 1aP1 (B), sema 2a (C), and sema 1aP1, sema 2a double mutant (D) 21-h embryos. (A) In sema 1aP1/+ control embryos Fas II tracts grow normally in the dorso-ventral axis. In both sema 1aP1 and sema 2a embryos Fas II tracts are affected (B and C) and the disruption is more severe in double mutants (D). (0.29 MB TIF) [file pbio.1000135.s006.tif]

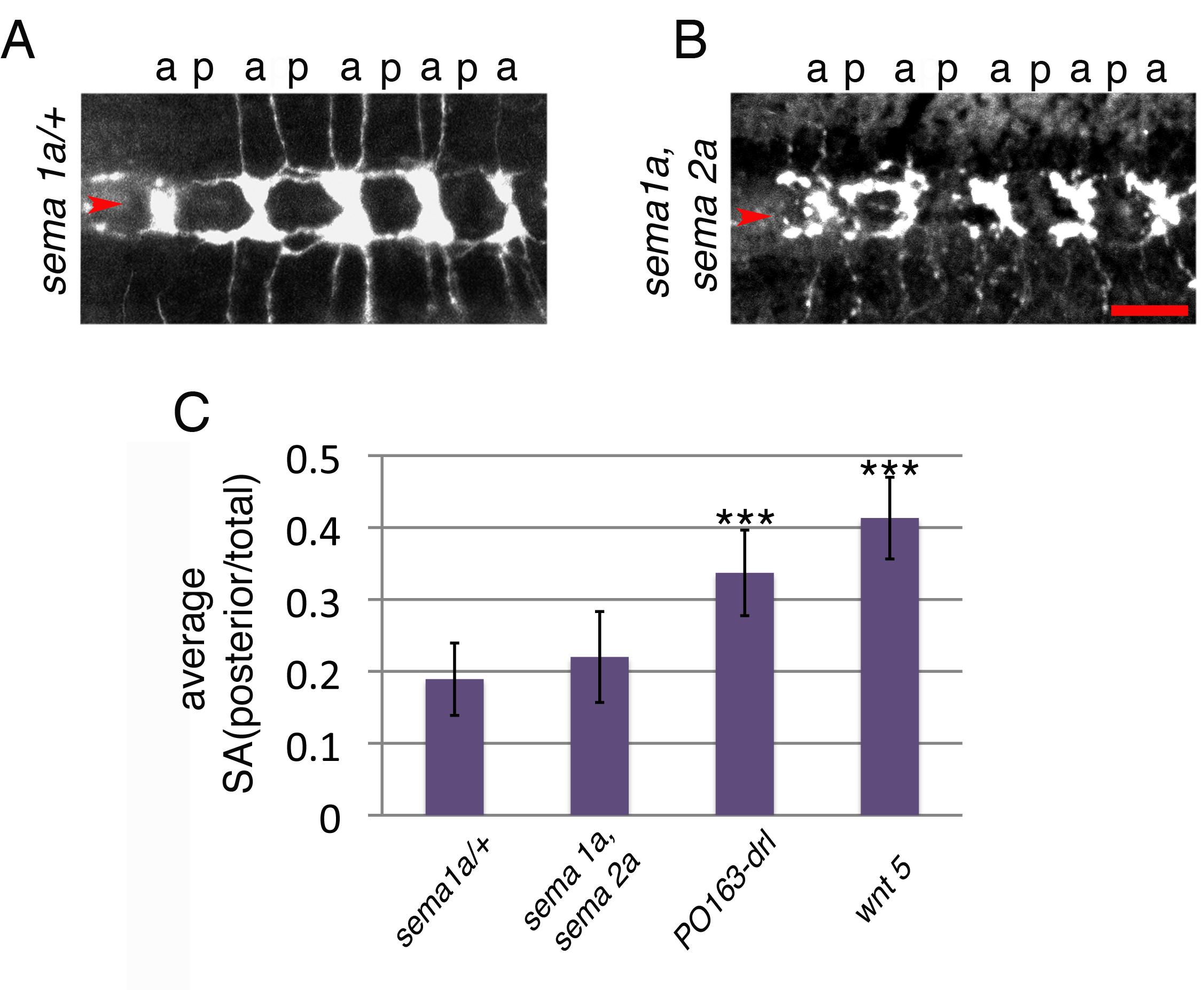

Supplement: Figure S7 — Role of Semas in patterning the antero-posterior axis of the neuropile. We assessed the potential role of Sema 1a and Sema 2a in controlling termination of class IV axons in the antero-posterior axis by analysing their projections in a top-down view of the neuropile in wild type and in sema 1a, sema 2a double mutants (see Figure S5A and S5B). We chose the sema 1a, sema 2a double mutant for this analysis, because it exhibited the strongest phenotypes in the dorso-ventral axis. (A and B) Projections of class IV axons labelled with ppkEGFP (white) in sema 1a/+; ppkEGFP control (A) and sema 1a, sema 2a; ppkEGFP (B), 21-h embryos. Images show projections of confocal z series of longitudinal sections of the VNC (from T1 to A4). Anterior left. Arrowheads show midline. a, anterior half of the segment; p, posterior half of the segment. Scale bar: 16 µm. (A) Top-down view of wild-type class IV projections in T1–A4. Wild-type class IV axons grow asymmetrically, within their normal ventral and medial termination domain, forming a thick anterior branch and very thin processes that extend posteriorly. (B) Top-down view of class IV projections in T1–A4 in sema 2a, sema 1a double mutants. Note that while the class IV terminals appear disorganised compared to wild type, they still appear asymmetric and largely confined to the anterior portion of the segment. We assume the observed disorganization is a consequence of the major defects in growth and termination in the dorsoventral axis. (C) Quantification of the average surface area occupied by class IV terminals in the posterior half of the hemisegment, relative to the total surface area covered by class IV terminals in a hemisegment [SA p/(p+a) = SA(posterior)/SA(posterior+anterior]. Quantification of SA p/(p+a) does not reveal a significant increase (p = 0.09; Student's t-test average SA p/(p+a) = 0.22; SD = 0.16; n = 50 hemisegments) for the double mutants with respect to wild-type embryos (average SA p/(p+a) = 0.19; SD = 0.1; n = [file pbio.1000135.s007.tif]

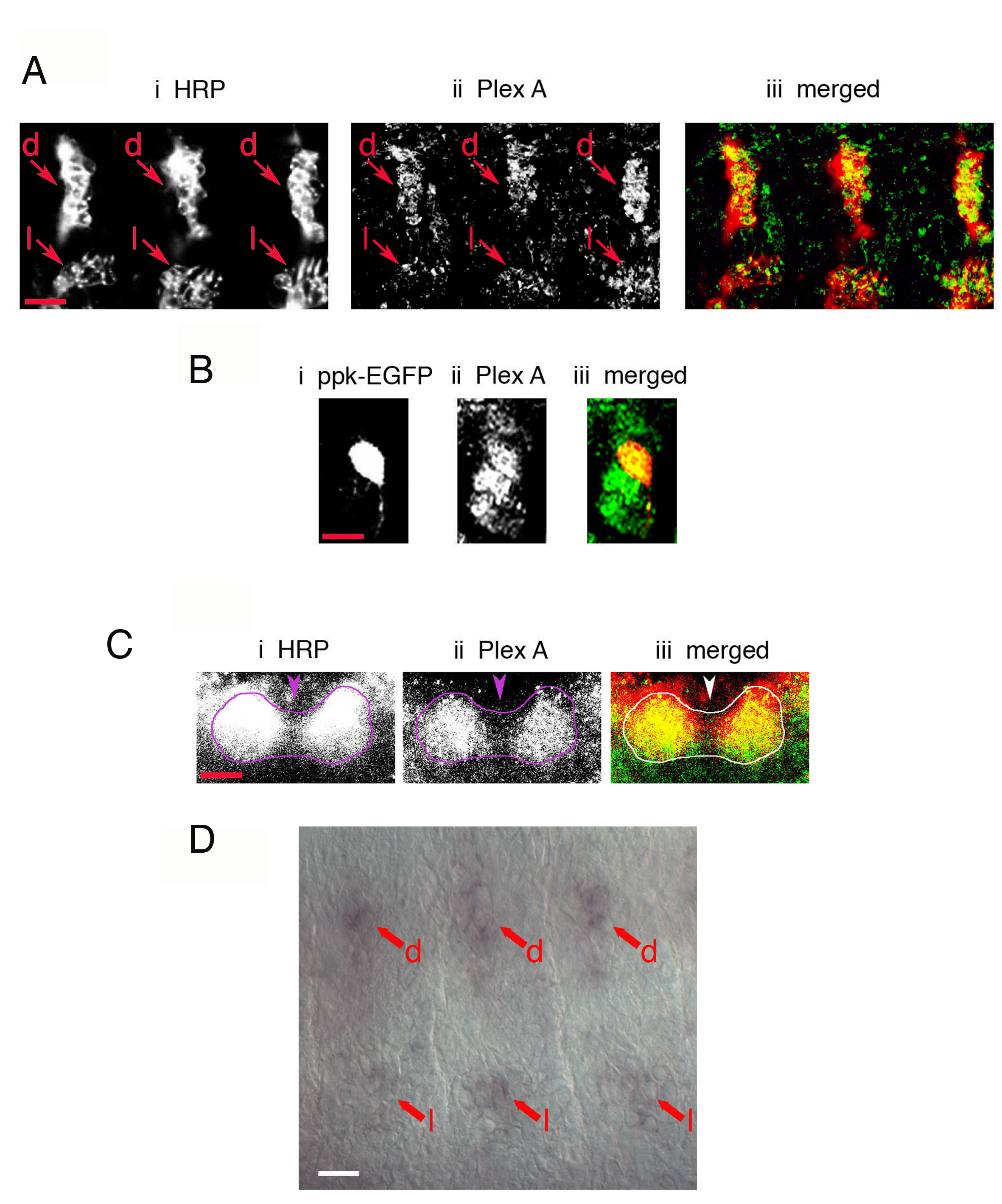

Supplement: Figure S8 — Plexin expression in sensory neurons (A, B, and D) and in the CNS (C). (A and B) Immunofluorescence visualisation of sensory neuron cell bodies labelled with antibody against horseradish peroxidase (HRP) (A) or PPK-EGFP (red) (in B) and Plex A (A and B) at 13-h AEL. Dorsal is up. (A) Plex A expression (white in ii and green in iii) in dorsal (d) and lateral (l) clusters of sensory neurons (white in i and red in iii). Strong Plex A expression is visible in sensory neuron cell bodies in both clusters. Scale bar: 15 µm. (B) Plex A protein (white in ii and green in iii) is strongly expressed in class IV md neuron cell bodies (white in i and red in iii). Scale bar: 10 µm. (C) Immunofluorescence visualisation of Plex A protein (white in ii and green in iii) in a transverse section of the neuropile labelled with HRP (white in i and red in iii) at 13-h AEL. Image shows a projection of a confocal z series of 1-mm thick transverse sections through abdominal segment A7. Dorsal is up. Arrowheads show the midline. Outlines indicate neuropile boundaries. Scale bar: 5 mm. (D) In situ hybridisation showing plex B mRNA expression in dorsal (d) and lateral (l) clusters of sensory neurons in the embryonic body wall. Dorsal is up. Scale bar: 20 µm. In situ hybridization protocol: DIG-labelled RNA antisense and sense probes were generated with the Ambion Megascript kit and DIG-UTP (purchased from Roche), following the manufacturer's instructions. In situ hybridization was performed according to a protocol kindly provided by Nipam Patel (University of California, Berkeley). DNA templates for in vitro transcription: DNA fragments were amplified by PCR with Primer1 (GCGCGCGTAATACGACTCACTATAGGG) and Primer2 (GCGCGCAATTAACCCTCACTAAAGGG) from pBluescript(SK)-PlexinB-CK00213 (AA142091) (EST, ∼1.7-kb insert) using the following key PCR parameters: annealing at 66°C, 5 min extension at 72°C, 30 cycles; Primer1 and Primer2 include the T7 and T3 promoter sequences. In vitro transcription: plex B: [file pbio.1000135.s008.tif]

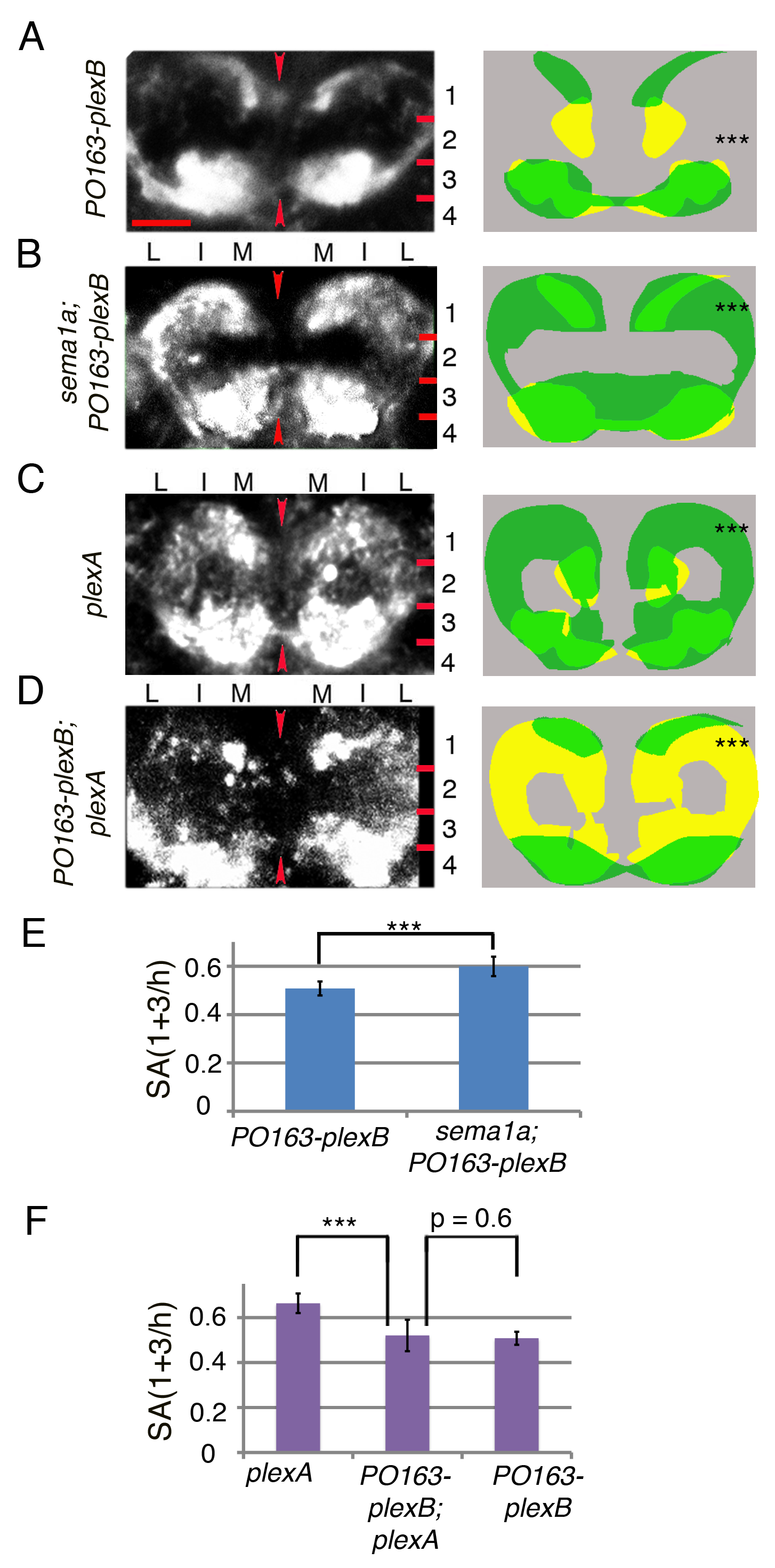

Supplement: Figure S9 — Plex B and Plex A prevent expansion of sensory terminals into regions with high Sema 1a levels. (A–D) Representative images of sensory terminals labelled with PO163GAL4, UAS-n-syb-GFP (white) in 21-h embryos (left) and diagrams showing patterns of sensory terminals superimposed for different genotypes (right). In all cases images show projections of a confocal z series of transverse sections through A7. Dorsal is up. Arrowheads show midline. White lines, layer boundaries. Numbers indicate layers: M, medial; I, intermediate; L, lateral domains. Scale bar: 10 µm. (A) Expressing Plex B in sensory neurons in a wild-type background results in exclusion of sensory neuron terminals from neuropile layer 2 (see also Figure 2C). Quantification of SA 2/h (SA 2/h = SA(layer 2)/[hemisegment surface area]) reveals a significant decrease (***, p = 4×10^−17^; Student's t-test; average SA 2/h = 0.003; SD = 0.006; n = 30 hemisegments) with respect to wild-type embryos (average SA 2/h = 0.06; SD = 0.02; n = 30 hemisegments). However, in these embryos, ectopic sensory terminals in layer 1 still remain largely excluded from intermediate and lateral portions of layer 1, which contain highest Sema 1a levels. Right: Diagram showing the pattern of Plex B expressing sensory terminals (green) superimposed on the wild-type pattern (yellow). (B) Expressing Plex B in sensory neurons in a sema 1a mutant background still excludes sensory terminals from neuropile layer 2. However, in these embryos ectopic sensory terminals invade the entire layer 1 and are no longer excluded from its lateral portions, which normally contain highest Sema 1a levels. This results in an overall increase in the surface area occupied by sensory neuron terminals in layers 1 and 3. Right: Diagram showing the patterns of Plex B expressing sensory terminals in sema 1a mutant (green) and wild-type (yellow) backgrounds, superimposed. Quantification reveals a significant increase in SA 1+3+4/h (SA 1+3+4/h = SA[layer 1+3+4]/[hemis [file pbio.1000135.s009.tif]

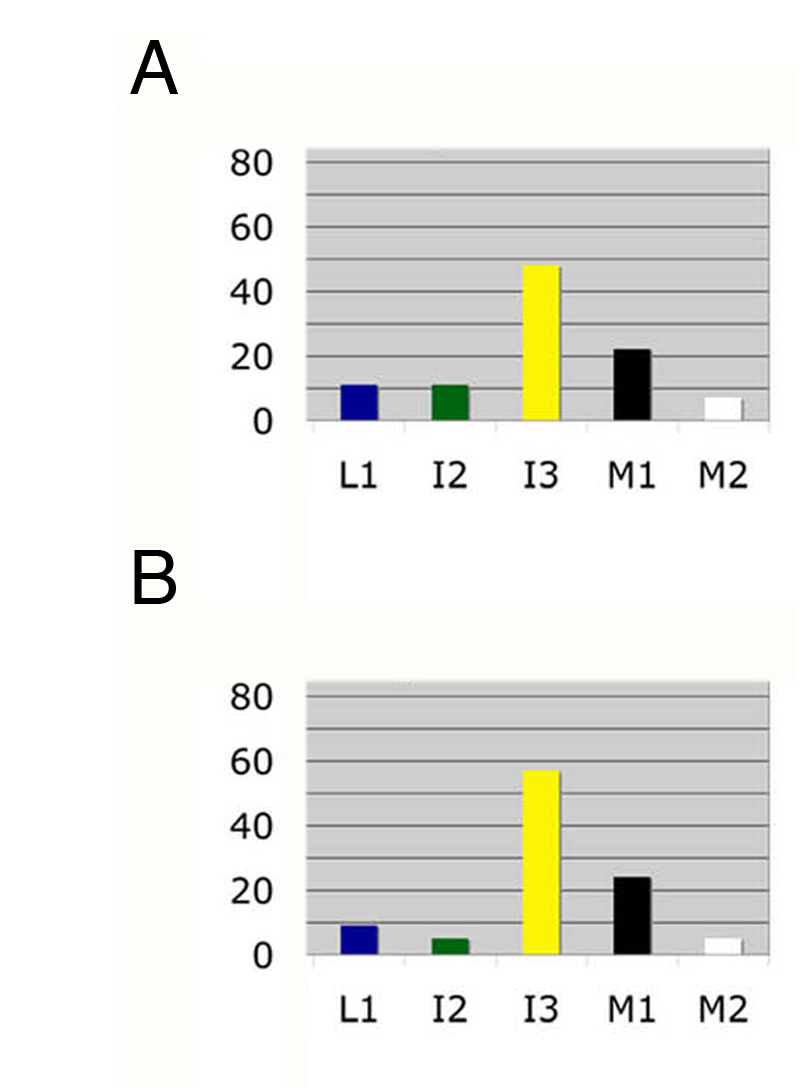

Supplement: Figure S10 — Fas II defects are not rescued by selective restoration of Plex B expression in sensory neurons. Graphs show percentage of segments (n = 175) in which L1 (blue), I2 (green), I3 (yellow), M1 (black), M2 (white) tracts project aberrantly. (A) In ppkEGFP; plexB embryos Fas II tracts are severely affected. (B) When Plex B expression is selectively restored in sensory neurons alone, in UAS-plexB;PO163GAL4,ppkEGFP;plexB embryos, Fas II tracts continue to exhibit the mutant phenotype. (0.19 MB TIF) [file pbio.1000135.s010.tif]
