# Supplementary material for: Positional Cues in the Drosophila Nerve Cord: Semaphorins Pattern the Dorso-Ventral Axis
Source: PLoS Biol. 2009 Jun 23;7(6):e1000135. doi: 10.1371/journal.pbio.1000135 (PMC2690435; doi:10.1371/journal.pbio.1000135)
Supplement: Table S1 — Results of the misexpression screen. We identified 11 genes (2.6%) that change the pattern of sensory terminals, without producing pronounced changes in neuron number or preventing sensory axons from reaching the CNS. In these experiments, sensory terminals shift independently of Fas II tracts, which remain in their wild-type position and relation to each other. Of the 11 genes, two produced specific shifts along the dorso-ventral axis. The table gives the list of 11 genes, which, when misexpressed in sensory neurons alone, produce specific shifts in the dorso-ventral, medio-lateral or antero-posterior axes. (0.04 MB DOC) [file pbio.1000135.s011.doc]

| **Over-expression Phenotype:** | **Gene:** |
| --- | --- |
| Shifts in the dorso-ventral axis | *plexA*  *plexB* |
| Shifts in the medio-lateral axis | *rhomboid-2*  *robo 2*  *robo*  *robo 3*  *egghead*  *wds*  *unc 5* |
| Alterations to pattern in the antero-posterior axis | *mew*  *PRL-1*  *ephrin* |

Table S1
